# Psychotherapeutic and Psychosocial Interventions with Unaccompanied Minors: A Scoping Review

**DOI:** 10.3390/healthcare11060918

**Published:** 2023-03-22

**Authors:** Irene Moutsou, Eugenie Georgaca, Theofilos Varaklis

**Affiliations:** School of Psychology, Aristotle University of Thessaloniki, 541 24 Thessaloniki, Greece

**Keywords:** unaccompanied minors, refugees, migrants, mental health, psychotherapy, psychosocial support

## Abstract

Unaccompanied minors (UAMs) are considered a particularly vulnerable population, facing severe threats regarding their physical and mental health. As their number has increased in recent years worldwide, research on mental health interventions has become necessary. The implementation of psychotherapeutic interventions has been documented, but psychosocial interventions seem to not have been consistently studied. In this review, we summarize the psychotherapeutic and psychosocial interventions with UAMs that have been studied up to now. Following the PRISMA guidelines for scoping reviews, we searched four databases and included studies and practice papers; there were no restrictions on publication date, geographical region, language, or method. We identified 46 studies on psychotherapeutic interventions and 16 studies on psychosocial interventions that met the inclusion criteria. Psychotherapeutic interventions were mainly based on cognitive behavioural, psychodynamic, narrative, art and transcultural approaches and aimed at improving UAMs’ trauma, mental health and wellbeing, as well as professionals’ skills and therapeutic protocols. Several studies showed promising results, with the cognitive behavioural approaches being the most researched. However, more research is needed in order to draw conclusions in terms of effectiveness. Psychosocial interventions followed various approaches and aimed at UAMs’ empowerment, wellbeing, support and integration, as well as at improving caregivers’ skills. Nonetheless, they seem not only very heterogeneous but also understudied, and we believe that a focus on them would be very useful. Methodological limitations and their implications for future research are discussed.

## 1. Introduction

According to the United Nations High Commissioner for Refugees (UNHCR) [1] (p. 5), “*an unaccompanied child is a person who is under the age of eighteen, unless, under the law applicable to the child, majority is attained earlier and who is separated from both parents and is not being cared for by an adult who by law or custom has responsibility to do so*”. Although the term “unaccompanied minor” (UAM) is often used in the literature as an equivalent to the term “unaccompanied refugee minor” (URM), we prefer to use the former, as it can include unaccompanied children of different legal statuses (e.g., undocumented migrants, asylum seekers, refugees). UAMs can be distinguished from *“separated children”, as the latter are “those separated from both parents, or from their previous legal or customary primary caregiver, but not necessarily from other relatives. These may, therefore, include children accompanied by other adult family members.*” [2] (p. 13).

It is estimated that 36.5 million (41%) of the 89.3 million forcibly displaced people at the end of 2021 were children below 18 years of age [3]. Furthermore, 17,200 (71%) of the 24,100 children who arrived in Europe between January and December 2021 were unaccompanied and separated. Most of them were boys, 15–17 years old [4]. Migration has been described as a traumatizing event related to separation and loss, loneliness, a lack of a sense of belonging, threats towards one’s identity, developmental regression and grief [5,6]. Unaccompanied minors (UAMs) are considered a particularly vulnerable group, as by definition, they are not accompanied by parents or carers and, thus, are more exposed to unsafe pre-, peri- and post-migration circumstances that pose severe threats to their physical and mental health [7,8].

Detention or death of the parents, as well as security and financial issues, lead families to the decision to let their children flee unaccompanied. Separation from the family, death of the parents or close relatives, exposure to armed conflict and threats to life are some of the main stressful events for UAMs. Poor living and sanitation conditions; lack of nutrition; health care and education; child labour; injuries; neglect; exploitation; physical and sexual abuse; adversities and violence during travel; post-migration challenges, such as racism, unemployment and poor living conditions in the host country, are additional traumatic and stressful experiences, due to which UAMs are considered a high-risk group [9]. The quality and quantity of stressful experiences have been found to be associated with depression, anxiety, post-traumatic stress syndrome (PTSS), post-traumatic stress disorder (PTSD), behavioural problems and other mental health issues, such as substance abuse [7,10]. Post-migration factors seem to be important determinants of mental health outcomes, and the prevalence of mental health problems in UAMs is higher compared to accompanied minors [10]. The mental health outcomes seem to be related to protective factors (e.g., communication with the family, social support, gender, cultural competence) [11], as well as to the current living circumstances (e.g., the type of accommodation [11,12] or facing legal difficulties concerning being granted asylum [7]).

Acculturation can be highly challenging and stressful for UAMs. However, it can also contribute to the development or strengthening of resilience while minors adopt several survival strategies [13]. Demographic, social, cultural, psychological and behavioural factors, as well as the quality of housing, seem to affect the acculturation process and acculturative stress [14,15].

Many institutions, organizations and research projects have focused on finding and applying effective mental health interventions for UAMs. According to the World Health Organization, four areas of intervention are of great importance for the wellbeing of refugees: (a) promoting social integration, (b) overcoming barriers to accessing mental health care, (c) facilitating engagement with services and (d) providing treatment for mental disorders [16]. Furthermore, a stepped approach for refugee minors has been recommended, starting from environmental-supportive factors, followed by short-term psychological group interventions focusing on psychoeducation and stress reduction, and then specialized mental health care, if needed [17]. Moreover, a holistic approach regarding the refugee minors’ experience, vulnerability and resilience, as well as professionals’ cultural awareness, have been considered important [18,19].

Various reviews on therapeutic and supportive interventions have been published. We found one practitioner [19] and two systematic reviews [20,21] (one of them including a meta-analysis [20]) that examined psychotherapy for refugee children with war-related trauma and/or mental distress, as well as a systematic [22] and a scoping review [23] examining psychotherapy specifically with UAMs. Several therapeutic approaches were reported, including cognitive behavioural therapy (CBT); trauma-focused cognitive behavioural therapy (TF-CBT); interpersonal therapy (IPT); narrative exposure therapy (NET); narrative exposure therapy for children (KidNET); eye movement desensitisation reprocessing (EMDR); testimonial psychotherapy; and systemic, transcultural, art and multimodal therapies. Despite the promising results identified, all the reviews suggested further research, as it was difficult to draw conclusions on the effectiveness of the interventions due to the methodological limitations of the studies. Furthermore, the exploration of other interventions (e.g., school-based programmes) was proposed. Similarly, a systematic review of psychosocial interventions for newly arrived adolescent refugees [24] found positive effects in the processing of trauma and forced migration, but the authors noted a lack of comprehensive, long-term and high-quality research in this field. Finally, another systematic review found group interventions effective for UAMs [25].

In our view, there is an important distinction between psychosocial support (PSS) and psychotherapy or counselling. PSS can be defined as “*the processes and actions that promote the holistic wellbeing of people in their social world. It includes support provided by family and friends. PSS can also be described as a process of facilitating resilience within individuals, families and communities. PSS aims to help individuals recover after a crisis has disrupted their lives and to enhance their ability to return to normality after experiencing adverse events*” [26]. On the other hand, psychotherapy and counselling refer to the communication of a trained professional (therapist/counsellor) and a client, aiming at assisting the latter in coping with their personal problems (emotional, behavioural, relational, etc.), seeking solutions and modifying their way of thinking accordingly [27,28]. Psychotherapy and counselling can be part of PSS; however, PSS is a much broader field, focusing on “*helping people connect with other family and community members, or helping them deal more effectively with personal challenges or practical problems*” [29]. International organizations that coordinate various residential and psychosocial programs have emphasized the importance of UAMs’ psychosocial support and offered general guidelines [30,31]. Thus, although not all UAMs are referred to psychotherapeutic or counselling services, the majority of those who are registered in state/asylum services and are placed in any kind of official accommodation programme receive PSS services.

A recent realist review [32] on the psychosocial care of UAMs underlined minors’ intersections of transitions of adolescence and migration, their needs for culture- and gender-sensitive psychosocial care and the undersupply of such care. It also discussed how the contexts and outcomes of UAMs’ psychosocial care were mediated by pre-, peri- and post-migratory stressors as well as care structures that were, in turn, influenced by overarching discourses and concepts.

Research on psychotherapeutic and PSS interventions is ongoing, as well as the need for effective psychosocial care practices for UAMs. The seven reviews that we identified on the subject offer insight into the research efforts and gaps regarding the minors’ therapeutic and psychosocial support. However, four of them were published five to 17 years ago [19,20,22,23], while the rest examined very specific aspects of the subject, namely interventions that were examined exclusively through quantitative studies [21], psychosocial interventions during the first year of arrival [24] and group interventions [25]. Therefore, to our knowledge, there is not a recent review that covers all research methods and types of intervention for both psychotherapy and PSS regarding the population of UAMs. We believe that an update on the research trends and gaps that would take into account and synthesize recent reviews while supplementing them with more information and providing a more complete picture could be useful. Thus, we consider it important to summarize and discuss both psychotherapeutic and PSS interventions for UAMs that have been studied up to now, focusing on their aims, findings and limitations, as well as what these indicate for future studies and for the support of UAMs.

### Aim of the Study

This scoping review aims to provide an overview of studies and clinical reports regarding psychotherapeutic and PSS interventions for UAMs. The data we summarize and present concern the type and aim of the intervention, the setting, the research method as well as the results and estimated effectiveness.

## 2. Methods

We followed the guidelines of the PRISMA Extension for Scoping Reviews [33]. Our study was registered on the Open Science Framework on 8 February 2023 (https://doi.org/10.17605/OSF.IO/SZ4VG).

### 2.1. Search Strategy

We chose four databases (Scopus, PsycArticles, Web of Science and PubMed) that seemed to be used more frequently on the subject and, to our knowledge, contain a great volume of the literature. The databases were initially searched on 5 December 2021. As six months passed from that date while we were still working on the paper, a supplementary search was conducted on 26 August 2022, following the guidelines of the *Cochrane Handbook for Systematic Reviews of Interventions* [34].

Due to the broad scope of our research question, namely which psychotherapeutic and psychosocial interventions for UAMs have been studied up to now regardless of method or specific approach, we decided to conduct an open search, including only terms about the population and the broader type of intervention, and therefore we did not use established search tools (e.g., PICO or SPIDER). Regarding the population, the search terms were “unaccompanied” and “minor”/“child”/“adolescent”/“young people” and “refugee”/“migrant”/“immigrant”. Regarding the psychotherapeutic interventions, the search terms were “therapy”/“psychotherapy”/“counselling”, while for the PSS interventions, the terms were “psychosocial”/“social” and “support”/“intervention”. On PubMed, some of these keywords were replaced by Medical Subject Headings (MeSH) terms. The search strategies were drafted by the first author and refined through team discussion with the second author and two other doctoral students. The final search strategy for Scopus and PubMed can be found in Table S1 and Table S2, respectively. The final search results were exported into RIS files and uploaded on the free web tool Rayyan, where duplicates were removed.

The electronic database search was supplemented by scanning relevant reviews. All papers identified in these reviews that fulfiled the study criteria were sought for inclusion, although not all were finally included due to lack of access to some of them. We opted to include papers included in previous reviews within our own review rather than move on from them because of differences in the inclusion criteria adopted by different reviews. The systematic review of Cowling and Anderson [21] was not scanned for the literature, as it was published while this paper was being written.

### 2.2. Inclusion and Exclusion Criteria

#### 2.2.1. Types of Papers and Studies

There was no limit regarding publication date, geographical location, language or ethnicity of the participants. We included both quantitative and qualitative research papers, as well as case studies, study protocols and practice papers that presented an intervention. Reviews were excluded, except when they presented a study by the authors in a separate section.

We decided to include not only research studies but also practice papers because our priority was to identify any kind of intervention with UAMs that has been applied and published without setting methodological criteria in terms of quality of research or empirically supported therapies (ESTs). We believe that professionals and researchers can benefit from or further develop ideas and initiatives that have not been studied in a research setting yet, especially in a field that is relatively new. Nevertheless, we present our observations on the studies’ methods and limitations.

#### 2.2.2. Subject

We included psychotherapeutic, counselling and PSS interventions. Interventions that were exclusively located in other disciplines, such as education, medicine or law, were excluded. Furthermore, we excluded papers that did not present at least one specific intervention, as well as studies in which the intervention was not exclusively designed for or applied to UAMs but was part of the routine practice of a facility or institution.

#### 2.2.3. Population

The participants had to be unaccompanied migrant, refugee, asylum-seeking or undocumented minors. They had to be less than 18 years old at the time of the intervention or when arriving at the host country. We did not set any limitations regarding sex or gender. We included research papers and clinical reports in which other populations were studied along with the UAMs as well as interventions designed for UAMs but applied to other populations (e.g., native minors). Finally, we included studies that concerned the training or supervision of professionals who offer psychotherapeutic or psychosocial services to UAMs.

### 2.3. Assessment of Studies

Following the method of a previous scoping review of psychotherapeutic interventions for UAMs [23], we assessed the studies based on the criteria of Chambless and Hollon [35] for ESTs and Kooistra et al. for good case series [36]. The assessment was conducted by the first and the third authors in consultation with the second author.

According to Chambless and Hollon [35], a treatment is “well established” when its efficacy is demonstrated (a) by at least two good between-group design experiments or (b) by a large series of single-case design experiments (*n* > 9) and (c) the experiments are conducted with treatment manuals, (d) the characteristics of the samples are clearly specified, and (e) the effects are demonstrated by at least two different investigators or investigating teams. In the case of a between-group design, the sample size per condition should be 25 to 30 participants. Moreover, in both (a) and (b) cases, the intervention should have been found statistically superior to a placebo or another treatment or equivalent to an established treatment. On the other hand, a treatment is “possibly efficacious” when (a) it is superior over a waiting-list control group; (b) it meets all criteria for a well-established treatment except demonstration by independent investigators; or (c) it consists of a small series of single-case design experiments (*n* > 3) meeting well-established treatment criteria regarding manual, sample and independent investigators.

According to Kooistra et al. [36], a good case series should (a) list and describe in detail its study population, the intervention and the primary outcome, (b) use only descriptive statistics for the analysis of results, and (c) thoroughly report the participants’ characteristics and the follow-up assessment data, without stating absolute conclusions. The blinding of outcome assessors is also recommended.

## 3. Results

**Figure 1:**
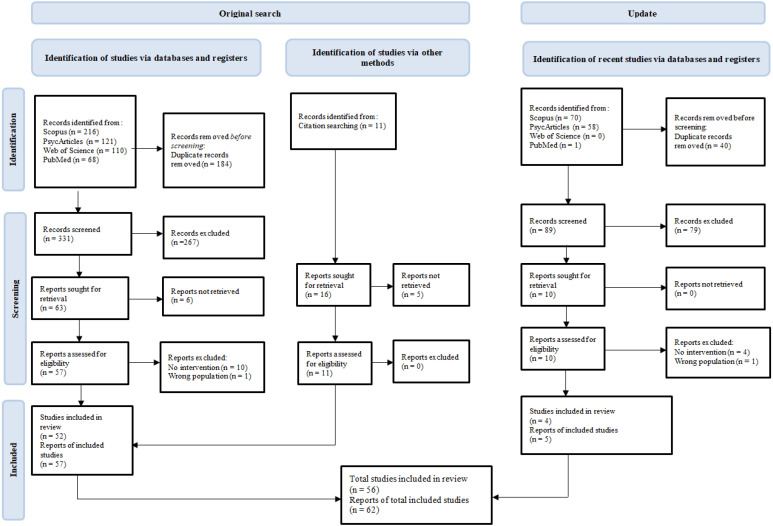
PRISMA flow chart.

The screening process was conducted by the authors and two other doctoral students using the free web tool Rayyan. Detailed information on the results of both searches is presented separately for greater transparency (see Figure 1). A data-charting form was jointly developed by the team.

A total of 526 titles and abstracts were originally identified. Of those, 11 were identified in a supplementary search by reading previous literature reviews. After duplicates were removed, 342 papers were subjected to title and abstract screening, resulting in 74 articles being selected and sought for retrieval. As 6 of them were not available even after sending a request to the authors, 68 papers were assessed for eligibility. A total of 11 articles were excluded, resulting in 57 included in this review. A supplementary search for the year 2021–2022 resulted in 129 titles and abstracts. After duplicate removal, 89 papers were subjected to title and abstract screening, resulting in 10 articles being sought for retrieval, 5 of which were found eligible. Of the total 62 papers included in the review, 46 were concerned with psychotherapy and 16 withvarious forms of PSS.

Over two-thirds (31) of the psychotherapy studies were published between 2017 and 2022. Most of them were conducted in Germany (13) and the UK (12), with small numbers in various European countries, Australia and the USA. The PSS studies were published between 2015 and 2021 and were conducted in various European and African countries, as well as Australia and the USA.

The studies included in this review were highly heterogeneous in terms of study design, methods and goals of the interventions. Detailed information is presented in Table S3 (Psychotherapeutic interventions) and Table S4 (PSS interventions).

### 3.1. Psychotherapeutic Interventions

Of the 46 papers that concern psychotherapy, 23 report CBT interventions [37,38,39,40,41,42,43,44,45,46,47,48,49,50,51,52,53,54,55,56,57,58,59], 6 transcultural interventions [60,61,62,63,64,65], 5 psychodynamic interventions [66,67,68,69,70], 2 NET interventions [71,72], 2 narrative interventions [73,74], 2 art interventions [75,76], 1 systemic intervention [77] and 5 examine various other approaches [78,79,80,81,82]. Furthermore, 12 studies followed a multimodal approach [45,46,51,58,59,67,68,69,70,78,81,82], yet we categorized them based on the therapeutic approach that seemed more prevalent, as they were very heterogeneous. Five of the papers concern further analysis [40,61,72], extra aspects [50] or systematization [75] of previously published studies [39,44,60,71,76]; thus, we do not regard them as distinct.

Regarding their aim, over two-thirds of the studies focused exclusively or partly on the alleviation of trauma-related symptoms [37,38,39,40,41,42,43,44,45,46,47,51,52,54,55,56,57,60,61,62,63,64,66,67,68,69,71,72,75,76,78,81]. Other aims were the improvement of mental health [48,49,53,58,59,63,65,73,74,75,77,78,79,80,82], promotion of wellbeing (including empowerment and resilience) [53,69,70,74,75,76,80], professionals’ training [50,58,60,61,78] and improvement of therapy [50]. Their duration and methods varied. All approaches considered building a working alliance, providing a safe environment and facilitating connectedness as important aspects of their intervention.

Most interventions took place in collaboration with community and welfare services [38,39,40,41,42,43,45,47,58,68,69,70,77,79,80,81], as well as outpatient clinics and hospitals [44,50,51,53,54,55,56,57,60,61,62,63,64,65,66,71,72,73]. Only two took place in a school setting [37,52] and eight in accommodation facilities, refugee camps and arrival centres [46,48,49,67,74,75,76,78,82]. All interventions were conducted by mental health professionals and trained therapists, except two that were conducted by trained teachers and educational staff [37,46]. Moreover, one intervention was conducted by staff members of accommodation facilities alongside mental health professionals, but their specialty was not defined [78].

Regarding the sample, the majority of the studies included mainly or exclusively male participants [37,38,39,40,41,44,45,46,47,48,49,51,53,54,56,58,59,60,61,62,63,64,65,66,67,68,69,70,71,72,73,74,75,76,77,78,79,80,81,82], adolescents or young adults [37,38,39,40,41,42,43,44,45,46,47,48,49,52,53,54,55,56,57,58,59,60,61,62,63,64,65,66,67,68,69,70,71,72,73,74,75,76,77,78,79,80,81,82] from countries of the Middle East and Africa [37,38,39,40,41,44,45,46,47,48,49,51,53,54,55,56,57,59,60,61,62,63,64,65,68,69,70,71,72,74,75,76,77,78,79,80,81,82] who were experiencing several forms of mental distress, including PTSD, depression and anxiety [37,38,39,40,41,42,43,44,45,46,47,51,52,54,55,56,57,58,59,60,61,62,63,64,65,66,67,68,69,70,71,72,73,77,78,79,80,81,82].

A summary of the interventions and their findings is presented below, grouped by their therapeutic approach.

#### 3.1.1. CBT-Based Interventions

Half of the CBT-based interventions concerned TF-CBT [38,39,40,41,42,43,44,47,50,52,54,55,56], while other studies examined CBT [37,48,57] and mindfulness [49], respectively. Six studies introduced multimodal interventions that combined CBT, TF-CBT or metacognitive therapy with various other approaches, namely NET/KidNET [45,51], EMDR [45], dialectical behaviour therapy (DBT) [46], time perspective therapy (TPT) [46], compassion focused therapy (CFT) [53], IPT for adolescents (IPT-A) [58] and pharmacotherapy [59]. Almost half of the interventions were group-based [37,38,39,40,41,43,46,47,48,49,53], while three more combined group sessions with individual sessions [42,52,58]. Four studies included sessions with the minors’ caregivers [43,47,52,55].

Common components of most interventions were psychoeducation, relaxation, construction of a trauma narrative, cognitive restructuring of unhelpful cognitions, learning coping strategies, as well as developing symptom management, emotional awareness and emotional regulation skills. Further focal points included addressing issues of physical health (e.g., diet, sleep, hygiene); issues related to power, racism and status; encouraging openness and acceptance; fostering social support; and facilitating participants in (re)gaining self-efficacy.

Significant findings were concerned with the decrease in PTSS, PTSD and trauma-related symptoms [37,38,39,40,44,45,47,51,54,55,56,57] as well as the improvement of depression [37,39,40,44,47,49,55], behavioural problems [37,44,53], negative affect [38,49], mental distress [41,46], anxiety [53,55] and physical complaints [44]. Moreover, increases in general wellbeing [48,53] and positive affect [49], as well as improvements in social relationships [51] and academic attendance or performance [51,52], were reported. The minors appeared to appreciate several aspects of their participation, such as a sense of safety, mutual support and connectedness [47,48,53,58]; learning new coping strategies [47,49,58]; normalization of their experiences [47]; and meaning making [47,48]. One study examining the feedback of professionals on TF-CBT found the treatment manual helpful, though a few adjustments were needed, and obstacles and cultural considerations were identified [50].

#### 3.1.2. Transcultural Interventions

All transcultural interventions were individual [60,61,62,63,64,65]; one of them included the minors’ social workers in the sessions [60,61]. The main focus was supporting the minors by taking into account their cultural affiliations, ways of thinking and migration experiences. Creativity was addressed as an important aspect of the sessions [60,61]. The interventions appeared to be helpful in enhancing the social workers’ cultural competence and their relationship with UAMs [60,61], developing UAMs’ identity and narrative of bicultural adolescence [60,61], as well as improving their self-esteem [64] and sense of belonging [65]. A positive feeling during the sessions [62] and the possibility of educational re-evaluation afterward [65] were also reported.

#### 3.1.3. Psychodynamic-Based Interventions

All psychodynamic-based interventions were individual [66,67,68,69], except for one that combined individual sessions with group meetings [70]. Moreover, four of them introduced a multimodal approach. Specifically, one study combined relational psychodynamic therapy with TF-CBT [67], and the rest integrated psychoanalytic therapy with aspects of the work of a community centre, such as a developmental approach, the adaptation and development after persecution and trauma (ADAPT) model, as well as daily activities and community meetings [68,69,70]. Processing issues concerning time, space, separation and loss, as well as feelings of uncertainty, vulnerability, fear, rage, hatred and guilt in a holding environment, were the main focus of these interventions. Restoring a sense of control was also reported as a primary therapeutic goal [67]. Concluding observations regarding outcome concerned sharing or a recalling of feelings [66,69], recovery of a positive sense of the self [66], a decrease in trauma-related symptoms and the improvement of social relationships [67], as well as alleviation of extreme protective defence mechanisms, emotional recovery and a hope of psychological survival [70].

#### 3.1.4. Narrative Exposure Therapy

Two papers presented a study of individual NET [71,72]. Its main components were diagnosis and psychoeducation, constructing a lifeline of key life experiences, narrating the lifeline, rereading the narration and signing the document. Improvement of the minors’ trauma-related symptoms was found, while the minors reported a sense of increased cohesion and meaning and considered the intervention helpful in understanding PTSD, integrating trauma in their life narrative, increasing their self-efficacy and expression, decreasing their symptoms and achieving post-traumatic growth.

#### 3.1.5. Narrative-Based Interventions

Of the two narrative-based studies, one consisted of individual sessions with the “Continuing Bonds” method [73], and one was a “Tree of Life” group intervention [74]. Re-narrating the minors’ stories was their common component. Discussions on culture, family expectations, resources and present difficulties were also reported. A feeling of connectedness and safety [73,74], as well as a positive change in how the minors perceived their problems and hardships [74], were observed.

#### 3.1.6. Art-Based Interventions

Two papers examined an art-based group intervention named expressive arts in transition (EXIT) [75,76]. It combined movement, dance, imagery and painting, focusing on connection and engagement, calming, efficacy, identity and hope. It was found to improve post-traumatic symptoms, life satisfaction and expected life satisfaction, but not general distress [75].

#### 3.1.7. Systemic Interventions

A group intervention based on systemic psychotherapy was found to decrease minors’ symptoms of depression and alexithymia and improve their self-esteem [77]. The sessions focused on the minors’ migration, family, identity, culture and acculturation stress, educational environment and integration.

#### 3.1.8. Other Interventions

Five studies examined various approaches, namely a phase-based approach to trauma care based on the guidelines of the Inter-Agency Standing Committee (IASC) for Mental Health Psychosocial Support (MHPSS) [78], photolanguage [79], psychodrama [80], a multimodal model of trauma and recovery [81] and an individual multimodal approach combining psychotherapeutic crisis intervention and ego-supportive approach [82], respectively. Their methods varied significantly. Findings and concluding observations concerned improvement in symptoms of mental distress and trauma [80,82], as well as in trust and communication [78], transformation and change [79], creation of a safe environment, empowerment and normalization of experiences [81].

### 3.2. Psychosocial Interventions

The 16 studies that concern PSS are very heterogeneous in terms of approach; thus, we can group them only based on their aim. Specifically, most of them focused on the minors’ wellbeing, empowerment and support [83,84,85,86,87,88,89,90,91,92,93]. Three studies aimed exclusively or partly at minors’ integration [88,94,95] and four aimed at the improvement of their caregivers’ skills [90,96,97,98]. One paper [85] consists of a more systematic examination of a previously published practice [86], so we do not consider it distinct. The studies examined, almost equally, individual [83,89,94,95], group [84,85,86,96,97,98] and mixed [87,88,90,91,92,93] interventions.

Regarding their setting, seven studies took place in collaboration with several kinds of community services, including a school [83,84,85,86,88,90,97,98]; six took place in refugee camps and residential facilities [87,89,91,92,93,96] and two in a laboratory [94,95]. The interventions of eight studies were conducted partly or exclusively by mental health professionals [85,86,88,89,91,92,96,98]. Interestingly, in eleven studies, several other professionals, volunteers and community members were involved. Specifically, three interventions were conducted by scientists of other specialties (computer, social, educational) [88,94,95,98], two by community members [84,87], one by trained volunteers [83] and two by various staff members of residential facilities [91,93], with or without mental health professionals. Finally, in one intervention, an artist has been involved [85,86], and one study concerned programmes that were run by several agencies and volunteer guardians [90].

The research samples were quite heterogeneous; however, in a significant number of studies, the participants were mainly male [83,84,85,86,87,88,89,91,97], adolescents or young adults [83,84,85,86,87,88,89,97] from countries of the Middle East and Africa [83,85,86,87,88,89,91,97]. Ten studies also included other populations apart from UAMs (e.g., native children; adult individuals; and families, mentors, group coordinators, personnel of residential facilities or volunteer guardians) [83,84,87,90,92,94,95,96,97,98].

A summary of the interventions and their findings is presented below, grouped by the aim that seemed primary.

#### 3.2.1. Wellbeing, Empowerment and Support

Interventions aiming at minors’ wellbeing, empowerment and support [83,84,85,86,87,88,89,90,91,92,93] used various creative methods and took place in several different settings. From an informal support group of Guatemalan former UAMs in the USA [84] to an art-based group named “The Suitcase Project” with unaccompanied children at a local school in South Africa [85,86] and a series of indigenous approaches for the support of UAMs in a refugee camp in Malawi [87], all interventions facilitated the participants’ self-expression and connection with their carers or counsellors in order to help them cope with traumatic events of the past, as well as with present difficulties and worries, and build their life in their new place of residence. A focus on the social and political aspects of their experience was also reported (e.g., by pointing out injustices and human rights) [89,92]. Moreover, a proposal for a multimodal residential programme in Portugal offered rich ideas concerning UAMs’ holistic support [93]. Findings and concluding observations refer to the minors’ increasing sense of safety and stability [87], improvement of their mental health symptoms and wellbeing [87,89,91], as well as of their view about themselves and their life (e.g., self-esteem, resilience, migration experiences) [83,84,85,86,92], facilitating their acculturation [84,88,90] and developing their personal narrative [84,85,86].

#### 3.2.2. Integration

Two interventions aimed to foster minors’ integration into their host society. The first focused on learning culture-related gestures based on social communication using robots [94,95]. The second reported a career guidance project based on the “life designing” paradigm of Savickas et al. [99] that combined group and individual meetings, aiming to help UAMs construct a decent life and find meaningful jobs [88]. Both appeared to be helpful in improving social interactions [94,95] and minors’ knowledge and interests regarding work, the labour market and professions [88], respectively.

#### 3.2.3. Improvement of Caregivers’ Skills

Interventions focusing on caregivers’ skills were similarly heterogeneous and concerned various types of caregivers, namely the staff of NGO services and facilities [96,98], foster carers [90,97] and the general host population [90]. The main goals were processing their concerns and perceptions [97,98], fostering their collaborative relationships and wellbeing [96], as well as helping their connection with the minors [90]. According to findings and concluding observations, through these interventions, foster carers expressed anxieties and misconceptions around caring for UAMs and appeared to become more open for further training [97], staff members experienced a decrease in their distress and an increase in their job satisfaction [96] and the host population seemed to approach UAMs [90]. Furthermore, helpful consultation strategies for staff working with UAMs were identified [98], and “playfulness” was considered a good means to improve communication and case management [96]. Finally, the minors were able to develop their autonomy [90].

### 3.3. Empirical Support of Interventions

Of the 21 CBT-based studies, over half were quantitative or mixed-method [37,38,39,40,41,42,43,44,45,46,47,48,49]. Furthermore, five of them were randomised controlled trials (RCTs) [37,39,40,41,46,48] and two RCT study protocols [42,43]. Similarly, research on NET [71], EXIT [75] and systemic psychotherapy [77] used partly or exclusively quantitative methods, although not RCTs. On the other hand, transcultural [60,61,62,63,64,65], psychodynamic [66,67,68,69,70] and narrative interventions [73,74], as well as the various other psychotherapeutic approaches [79,80,81,82], were studied mainly through qualitative or case studies and practice papers. Of the 16 PSS studies, four were quantitative and mixed-method [83,94,95,96] (one of them controlled [83]), four qualitative [84,85,87,97] and the rest were case studies [88,89,90,98] and practice papers [76,91,92,93].

In total, four quantitative and two mixed-method studies examining CBT [37,48], TF-CBT [39,40,41], multimodal [46], art-based [75] and mentoring [83] interventions found them effective compared to a control condition regarding a decrease in trauma-related symptoms and mental distress [37,38,39,40,41,46,75], as well as an increase of life satisfaction and wellbeing [75,83]. Apart from two [75,83], all studies were RCTs. However, none of them fulfil the criteria for a “well-established” or even a “possibly efficacious” intervention. Specifically, in three of them, the random allocation in groups was partial [41,48,75], while in all studies, participants were not randomly recruited. Furthermore, in one study UAMs were only a small part of the participants [37], and in all studies except two [39,40,75], the sample size was less than 25–30 participants per condition. Additionally, most or all participants were male, and in four out of six studies [39,40,41,46,48], they came mainly from one or two countries. Thus, the generalizability of the findings is limited. The findings of three studies are based solely on self-report measures [37,48,75], while all studies include at least one measure for which data on cultural validity are conflicting or not available. The external assessment used in one study [46] contradicts its self-report findings, as it shows significant improvement both in intervention and in the control group regarding mental distress. Finally, in two of these studies [48,83], there was no follow-up assessment, while in three [37,40,41], significant rates of loss during the follow-up were reported.

The uncontrolled quantitative or mixed-method studies [38,44,45,47,49,71,78,94,95,96], as well as the qualitative [50,60,61,72,84,85,87,96] and case studies or series [51,52,53,54,55,56,57,62,63,64,65,66,67,68,69,73,74,79,80,81,88,89,90,98] and the practice papers [58,59,70,76,82,86,91,92,93], presented similar limitations regarding the size and characteristics of the sample and attrition rates, as well as the lack of random recruitment, control group, standardized and culturally validated measures and follow-up assessment. Furthermore, a great number of them did not present a detailed description of their therapeutic methods, making their replication difficult [51,62,63,64,65,66,67,68,69,70,73,79,80,81,83,84,87,88,89,90,91,92,97,98]. Finally, in almost half of the studies, the effects of the intervention were not demonstrated by at least two different investigators. Thus, their reliability is limited [37,51,52,53,55,56,57,62,63,64,65,67,68,69,74,76,79,80,81,82,84,85,86,88,89,94,95,97,98].

## 4. Discussion

### 4.1. Overview and Evaluation of Interventions for UAMs

In our literature search, we found 46 studies of psychotherapeutic interventions and 16 studies of PSS interventions. Both types aimed at addressing UAMs’ difficulties and offering them support, with psychotherapeutic interventions focusing mainly on improving UAMs’ mental health and trauma, and PSS targeting mainly their wellbeing and empowerment.

Several different psychotherapeutic approaches have been studied, with CBT being the most researched. Among other approaches were psychodynamic, transcultural, NET, narrative, art, and systemic interventions. Furthermore, a significant number of studies introduced multimodal approaches presenting promising results. As previously mentioned, a common ground for all interventions was a focus on providing a safe environment and building a good connection.

CBT interventions [38,39,40,41,42,43,44,45,46,47,48,49,50,51,52,53,54,55,56,57,58,59] presented various significant results, regarding decreasing trauma-related symptoms and other forms of mental distress, as well as improving wellbeing, positive affect, social relationships and academic attendance or performance. Moreover, the minors evaluated their therapeutic experience positively regarding their symptoms, learned coping strategies and participation. Moreover, group support and the normalization of experiences were reported as important outcomes. This is in line with the findings of a recent review pointing out that minors prefer attending group interventions as they value social interactions [100].

Although none of the CBT studies reached the Chambless and Hollon [35] criteria for “well-established” or “possibly efficacious” ESTs due to significant limitations, it is obvious that there has been a systematic and noteworthy effort and improvement in research methods since the previous scoping review [23] on the subject. The increase in RCTs and controlled studies is indicative of that. Thus, as Cowling and Anderson [21] also pointed out, the findings are very promising; nevertheless, it is still difficult to draw conclusions in terms of effectiveness.

The studies of other psychotherapeutic approaches also presented various creative interventions with promising findings or observations. From the exploration of culture and migration in transcultural sessions [60,61,62,63,64,65] to the processing of difficult feelings in psychodynamic sessions [66,67,68,69,70], and from the construction of a therapeutic life narrative in NET [71,72] and narrative-based interventions [73,74] to the self-expression through art and movement with EXIT [75,76], all approaches appeared to have something beneficial to offer. Similarly to CBT, findings and concluding observations point out the decrease in trauma-related symptoms and other forms of mental distress, as well as the improvement of self-esteem, wellbeing and social relationships, along with positive feedback regarding a sense of safety and connectedness during the sessions. However, apart from EXIT [75], none of the other approaches were studied through a controlled design. Moreover, the small number of studies of each approach, combined with the limitations that are common to most of them (e.g., small sample size and non-heterogeneous sample), impedes drawing any conclusions on their effectiveness.

The PSS interventions [83,84,85,86,87,88,89,90,91,92,93,94,95,96,97,98] published varied significantly in terms of approach. However, they shared some common aims, namely improving the wellbeing, empowerment and integration of the minors and their caregivers’ skills. Many of their findings, such as providing a sense of safety, improving mental health and self-esteem and developing a personal narrative, were similar to those of psychotherapeutic interventions. Nonetheless, the facilitation of minors’ acculturation and integration, as well as processing concerns and difficulties of various caregivers and professionals who support them, were addressed more distinctly in PSS interventions.

In terms of effectiveness, it is again not possible to draw conclusions due to significant limitations. Characteristically, only one study [83] examining a mentoring program used a controlled design, and yet it presented several methodological issues (e.g., lack of follow-up assessment).

### 4.2. Comparing Psychotherapeutic and Psychosocial Interventions

Both psychotherapy and psychosocial care have been considered of great importance for the support of UAMs, while the approaches, methods, barriers and policies around them are still under discussion and research. However, as our results indicate, psychotherapeutic interventions have been studied significantly more than PSS. Furthermore, comparing the two, we observe some noteworthy differences regarding their aims, participants, setting and country of origin. Psychotherapeutic research is mainly aimed at treating minors who experienced trauma-related symptoms, often fulfiling the diagnostic criteria for PTSS or PTSD. The interventions took place mainly in community services by mental health professionals, and all studies were conducted in high-income countries, with the vast majority coming from western, central and northern Europe, especially Germany and the UK. On the contrary, PSS research aimed mainly at empowering minors and improving their wellbeing, regardless of pre-existing trauma or mental distress symptoms. It also appeared to address their integration and their caregivers’ skills and needs more explicitly. Although most PSS interventions took place in community services as well, a greater proportion compared to the psychotherapeutic ones took place in residential facilities. Moreover, they were conducted not only by mental health professionals but also by several other professionals, residential staff members, volunteers and community members. Finally, a significant number of these studies originated from southern Europe and low-income countries of Africa.

By definition, PSS is a broader and multidisciplinary field that addresses a spectrum of needs ranging from material and practical to mental health and emotional and, thus, can have a preventive character [27,30]. On the other hand, psychotherapy mainly concerns people who are struggling with various forms of mental distress and are willing to engage in talking therapy with trained mental health professionals [27]. It is possible that in first-arrival countries (e.g., southern European [101]) or regions of Africa that host many internally displaced children [102], more basic, holistic or sometimes informal forms of support are developed as an initial response to a very heterogeneous population with various needs. Supportive interventions by NGOs [91] or community members [87] in refugee camps, integration and empowerment projects in residential facilities [93,96] and career guidance [88] are typical examples. This is in line with the IASC guidelines for mental health and PSS in emergency settings [103] that address multidimensional support and prioritize the fulfilment of basic needs, not only for people’s survival but also for restoring their sense of dignity.

Nevertheless, it seems that after the so-called “refugee crisis” in 2015, when more than a million forcibly displaced individuals arrived in Europe seeking asylum [101], the western scientific community has prioritized the treatment of severe mental distress and trauma caused by migration adversities by trying and adapting established therapies. Although most studies showed promising or at least interesting results, we believe that broadening the scope of such research in terms of aims, approach, setting and method can be useful and that more PSS research is needed.

As Demazure et al. [100] have pointed out, mental health is more than the presence or absence of psychopathological symptoms. Thus, studies on interventions that focus more on general wellbeing, integration and strengths and acknowledge the weight of anti-migratory policies on minors’ mental health and life would possibly be very helpful. Many minors are not referred to mental health services; they might be unwilling to use such services, whereas the availability of the services may be limited [104,105,106,107]. Language, stigma, low awareness and heterogeneous help-seeking behaviours have been addressed as the main barriers [108,109]. Nonetheless, UAMs try to cope with various pre-, peri-, and post-migratory challenges that are very difficult and stressful [7,110]. Moreover, research on minors’ views around mental health and professionals [100,111] has shown that they prioritize the fulfilment of their material, legal and educational needs and often do not trust mental health professionals and the process of talk therapy since, to some, it seems slow, past-oriented, intrusive, painful, stigmatizing, vain or very different from their coping strategies and cultural customs. They appear to prefer activity-based group interventions and talking about their daily problems [100].

From the studies included in this review, we conclude that various psychotherapeutic approaches take into account these needs (e.g., TF-CBT or EXIT). However, PSS interventions conducted by various kinds of professionals or community members, combining empowering and practical support, in residential, school or other community settings may respond to these needs more easily and for a greater percentage of minors. By this, we do not imply that current research on psychotherapeutic interventions is not useful; we rather want to point out the significance of examining other ways as well, based on the specific needs and circumstances of the population. Moreover, such research can be very helpful for the professionals and caregivers that support UAMs on a daily basis and are not mental health or psychotherapy experts (e.g., educators, mentors, residential staff members and foster carers).

### 4.3. Constraints and Limitations of the Studies

Another consideration of ours concerns the persisting difficulties in conducting methodologically sound studies that can offer trustworthy findings on the effectiveness of interventions. As we have seen, research with UAMs can be very complex and linked to several methodological and ethical issues [112,113].

Access to potential participants may be restricted due to statutory obstacles [113] or the population’s hesitance in engaging with mental health services and professionals [100]. Furthermore, dropouts may occur for various reasons, including social policies that affect minors’ legal status and place of residence [7] and the divergence between mental health professionals’ perceptions and minors’ expectations or priorities [100]. UAMs’ living circumstances and the policies involved vary across countries, and the diversity of the local institutional responses, as well as the structural violence of migration management, have grave effects on them [113,114,115,116]. Policies that promote residential and legal stability and safety could probably be beneficial. However, this seems a great challenge when taking into account the conflict between the humanitarian approach and restrictive migration policies [116,117,118,119]. Nevertheless, acknowledging and reporting the sociopolitical climate of the host country [110], as well as examining and respecting UAMs’ needs [100], can be helpful in contextualizing the minors’ experience and the methodological limitations.

Another issue is that most interventions are studied with a mainly or exclusively male sample. This limitation cannot be avoided, as female minors do not migrate unaccompanied that often, probably because of fears of sexual exploitation and abuse, and thus male UAMs are significantly more [4]. In our view, researchers should acknowledge that their findings concern male minors, not as a “limitation” but as the initial focus of their studies. Of course, research with female UAMs should be conducted as well, but given their small numbers, it might be better to test interventions through well-designed case series rather than recruiting just a few of them in controlled quantitative studies where the vast majority of participants are male. As Ulrich et al. [32] have pointed out, psychosocial care is male-specific or gender non-sensitive, and thus, the special challenges and needs of female UAMs are not sufficiently addressed and confronted [120]. Gender-specific studies could solve this issue.

The same viewpoint could stand in the case of ethnicities as well. The majority of UAMs come from certain areas or countries (e.g., Afghanistan) because, currently, these countries happen to be in a belligerent state or face other kinds of extreme adversities. Instead of perceiving unaccompanied minors as a unified group of people with similar characteristics [113] and trying to test interventions with multi-ethnicity samples, where some ethnicities are always overrepresented and others underrepresented, researchers could adapt their method based on the participants that they can or want to recruit each time (e.g., larger studies for larger ethnic groups and case series for smaller ethnic groups) and acknowledge their ethnic target group from the beginning. This can offer a clearer view of cultural similarities and differences as well as of culturally-specific needs and viewpoints regarding supportive interventions.

Finally, in our view, ethical issues are of great importance. As Chase et al. [113] point out, research is always influenced by our beliefs, assumptions and expectations. Thus, we actively participate in the construction of UAM’s definitions, characteristics, vulnerabilities, resiliences and needs through our discourses and methods [32,113]. Furthermore, we often do so by applying our western ways of understanding and treating mental distress to them, while power and status inequalities and differences between researchers and participants may affect the latter’s participation and the findings. Social policies and legislation that determine the minors’ future based on their experiences and vulnerabilities may also influence us or pose ethical dilemmas in research. Thus, it is crucial to reflect on our own position as researchers by acknowledging all these issues while designing and testing interventions [113]. This kind of reflection could also reduce the gap between professionals’ and UAMs’ views on mental health and distress and, therefore, improve their relationship and collaboration. Lems et al. [121] suggest more open and exploratory methods (e.g., ethnographic research) in order to allow richer narratives, which are not limited to the migratory experience that researchers, therapists and authorities expect to listen to. Thus, further qualitative research may be an essential preliminary step to the design and testing of effective interventions.

### 4.4. Implications for Future Studies

Based on our findings and the aforementioned observations, we suggest that research on psychotherapy for UAMs should focus more on general wellbeing, integration and strengths, along with the trauma-related symptoms, while more explicitly acknowledging the sociopolitical context of migration. Taking into account our cultural influences and how these shape our methods in research and therapy, as well as the minors’ views and needs, is essential. Further research on each therapeutic approach is needed in order to reach conclusions on their effectiveness. Moreover, we believe that more research on PSS is of great importance for improving more holistic care services that concern a greater percentage of UAMs and may have a preventive character. Gender- and ethnicity-specific studies could offer a richer understanding of minors’ needs and therapeutic experience, adapting the research method according to each population’s size. The same could possibly stand for other significant aspects of identity, such as sexual orientation, as well as other genders apart from female and male, always with respect to the participants’ views and understandings. Finally, further qualitative research could help in designing more culturally appropriate and effective interventions.

### 4.5. Limitations of the Review

Our review presents some limitations. Firstly, our last literature search was conducted in August 2022; thus, we did not include more recent studies. Furthermore, part of the literature that we found through previous reviews was not included because of a lack of access. Additionally, as our inclusion criteria were quite open, very heterogeneous studies and papers were included, posing challenges in their comparison. Finally, we did not conduct a quality assessment of the studies based on standardized measures that could probably offer a clearer and more complete evaluation of their methods.

## 5. Conclusions

Our literature search resulted in 62 papers; 46 concerned studies on psychotherapeutic interventions and 16 were studies on PSS interventions. Based on our findings, several studies on psychotherapeutic interventions have shown promising results concerning improving UAMs’ mental health and wellbeing. However, more research is needed in order to draw conclusions regarding their effectiveness. PSS interventions seem very heterogeneous and understudied, and we believe that intensifying work on them would be useful for the wellbeing and integration of UAMs as well as the support of their caregivers. Methodological limitations lead us to the conclusion that adaptations to research designs are needed, whereas the acknowledgement of the sociopolitical circumstances in the host countries as well as of the researchers’ position seems essential. Finally, further use of qualitative methods could offer a richer understanding of minors’ needs and, thus, lead to designing and testing more culturally appropriate interventions.

## Data Availability

The data presented in this study have been submitted to the journal and are also available on the Open Science Framework (https://doi.org/10.17605/OSF.IO/YDQ3T).

## Supplementary Materials

The following supporting information can be downloaded at: https://www.mdpi.com/article/10.3390/healthcare11060918/s1, Table S1: Detailed search strategy used for Scopus; Table S2: Detailed search strategy used for PubMed; Table S3: Overview of the 46 studies on psychotherapeutic interventions; Table S4: Overview of the 16 studies on PSS interventions.
