# Supplementary material for: Psychotherapeutic and Psychosocial Interventions with Unaccompanied Minors: A Scoping Review
Source: Healthcare (Basel). 2023 Mar 22;11(6):918. doi: 10.3390/healthcare11060918 (PMC10048295; doi:10.3390/healthcare11060918)
Supplement: Supplementary file 1 [file healthcare-11-00918-s001.zip › Table S1.pdf]

Table S1. *Detailed search strategy used for Scopus.*

**Database**	**Scopus**
**Date**	**05/12/2021 and 26/08/2022**
**Strategy**	#1 AND #2 AND (#3 OR #4)
#1<br>**Concept:** Unaccompanied Minor	unaccompanied AND (minor* OR child* OR adolescent*)
#2<br>**Concept:** Migrant	refugee* OR migrant* OR immigrant*
#3<br>**Concept:** Psychotherapy	therap* OR psychotherap* OR counsel*
#4<br>**Concept:** Psychosocial Support	(psychosocial OR social) AND (support* OR intervention*)
