# Supplementary material for: Psychotherapeutic and Psychosocial Interventions with Unaccompanied Minors: A Scoping Review
Source: Healthcare (Basel). 2023 Mar 22;11(6):918. doi: 10.3390/healthcare11060918 (PMC10048295; doi:10.3390/healthcare11060918)
Supplement: Supplementary file 1 [file healthcare-11-00918-s001.zip › Table S2.pdf]

Table S2. *Detailed search strategy used for PubMed.*

**Database**	**PubMed**
**Date**	**05/12/2021 and 26/08/2022**
**Strategy**	#1 AND #2 AND #3 AND (#4 OR #5)
#1<br>**Concept:** Unaccompanied	unaccompanied [tw]
#2<br>**Concept:** Minor	"Minors" [MeSH] OR "Child" [MeSH] OR "Adolescent" [MeSH]
#3<br>**Concept:** Migrant	"Refugees" [MeSH] OR "Transients and Migrants" [MeSH] OR "Emigrants and Immigrants" [MeSH] OR "Undocumented Immigrants" [MeSH]
#4<br>**Concept:** Psychotherapy	"Therapeutics" [MeSH] OR "Psychotherapy" [MeSH] OR "Counseling" [MeSH] OR "Psychological Techniques" [MeSH] OR "Mental Health Recovery" [MeSH]
#5<br>**Concept:** Psychosocial Support	"Psychosocial Interventions" [MeSH] OR "Psychosocial Support Systems" [MeSH] OR "Social Support" [MeSH]
