# Supplementary material for: Psychotherapeutic and Psychosocial Interventions with Unaccompanied Minors: A Scoping Review
Source: Healthcare (Basel). 2023 Mar 22;11(6):918. doi: 10.3390/healthcare11060918 (PMC10048295; doi:10.3390/healthcare11060918)
Supplement: Supplementary file 1 [file healthcare-11-00918-s001.zip › Table S3.pdf]

Table S3. *Overview of the 46 studies on psychotherapeutic interventions*

Author(s), Year, Country	Type of paper/study	Type of intervention	Aim of intervention	Setting	Participants	Measures (if any)	Results (if any)	Limitations
Quantitative studies								
		**CBT-based interventions**						
Ehntholt et al. (2005), UK	RCT	School-based CBT<br>Manualized<br>Group<br>6 weekly sessions	Trauma improvement	Secondary schools<br>Sessions conducted by EMAG teachers	26 asylum-seeking/ refugee children (9 F)<br>CBT: n=15 (2 UAMs)<br>Control: n=11 (4 UAMs)<br>11-15 years old<br>Kosovo, Sierra Leone, Turkey (Kurdish), Afghanistan, Somalia<br>Psychological and behavioural difficulties	R-IES, DSRS<br>RCMAS, WTQ<br>SDQ	Significant decrease in PTSD**, intrusive symptoms**, behavioural difficulties* and emotional symptoms*	Small sample size<br>Mainly male participants<br>Not randomly recruited<br>Limited generalizability<br>Loss to follow-up<br>Only self-report measures<br>R-IES and DSRS not culturally validated<br>Conflicting data on cultural validity of SDQ<br>No comparison with placebo or another intervention<br>Effects not demonstrated by different investigators
Pfeiffer & Goldbeck (2017), Germany	Uncontrolled pilot study	"Mein Weg"<br>Trauma Focused Intervention<br>Manualized<br>Group<br>6 weekly sessions	Trauma improvement	Collaboration with child welfare agencies<br>Sessions conducted by trained social workers	29 UAMs (M)<br>14-18 years old<br>Mainly Afghanistan<br>Mild to moderate PTSS	CATS	Significant decrease in PTSS**, re-experiencing**, avoidance*, negative alterations in cognition and mood**	Small sample size<br>Only male participants<br>Approx. 50% from one country<br>Strict inclusion criteria<br>Not randomly recruited<br>Limited generalizability<br>Only self-report PTSS measures<br>Conflicting data on cultural validity of CATS<br>Only analysis of complete data<br>No control group<br>No comparison with placebo or another intervention<br>No independent intervention assessment<br>No follow-up
Pfeiffer et al. (2018), Germany	RCT	"Mein Weg"<br>Trauma Focused Intervention<br>Manualized<br>Group<br>6 weekly sessions	Trauma improvement	Collaboration with child and adolescent welfare programmes<br>Sessions conducted by trained social workers	Mein Weg: n=47 (UAMs, 3F)<br>37 attended all sessions<br>Usual Care: n=49 (48 UAMs, 1 AM, 4F)<br>45 attended all sessions<br>13-19 years old<br>Middle East, Africa<br>At least moderately severe PTSS	CATS-Self<br>CATS-Care<br>PHQ-8, CPTCI-S<br>Socio-demographic factors	Significant improvement of self-reported PTSS** and depression**, but not of caregiver-reported symptoms and self-reported dysfunctional posttraumatic cognitions	Mainly male participants<br>Approx. 50% from one country<br>Strict inclusion criteria<br>Not randomly recruited<br>Limited generalizability<br>Spill-over effects within the participating sites<br>Possible bias of social workers in Usual Care group, due to training<br>Intervention fidelity based solely on self-reports<br>Conflicting data on cultural validity

								of CATS<br>Further follow-up needed
Pfeiffer et al. (2019), Germany	RCT<br>Secondary analysis of Pfeiffer et al. (2018) study	"Mein Weg"<br>Trauma Focused Intervention<br>Manualized<br>Group<br>6 weekly sessions	Trauma improvement	Collaboration with child welfare agencies<br>Sessions conducted by trained social workers	Mein Weg: 47 UAMs(3F)<br>37 attended all sessions<br>14-19 years old<br>Middle East, Africa<br>At least moderately severe PTSS	CATS-Self<br>CATS-Care<br>PHQ-8, CPTCI-S<br>Socio-demographic factors	Country of origin significant predictor of change in PTSS*, baseline levels of depression significant predictor of change in depression***<br>Sustainability of treatment effects in self-reported PTSS pre to post intervention*** and post to 3-month follow up*, and depression pre to post intervention***	Mainly male participants<br>Approx. 50% of the sample from one country<br>Strict inclusion criteria<br>Not randomly recruited<br>Limited generalizability<br>Conflicting data on cultural validity of CATS<br>No independent intervention assessment<br>Loss to follow-up<br>No long-term follow-up
Rondung et al. (2022), Sweden	RCT pilot study	TF-CBT<br>Teaching recovery techniques<br>Manualized<br>Group<br>7 weekly sessions	Trauma improvement	Collaboration between two Swedish universities<br>Recruitment from community services<br>Sessions conducted by TRT-trained research team members	15 UAMs (2F)<br>TRT: n=14<br>6 attended ≥ 4 sessions<br>Waitlist control: n=1<br>16-20 years old<br>Afghanistan, Eritrea<br>PTSD	CRIES-13, GSE<br>PHQ-9, GAD-7<br>CLS, RTHC, CHU-9D, TiC-P, C-SSRS	Reduction of mental health symptomatology, but not improvement in self-efficacy (descriptive data)	Recruitment difficulties<br>Small sample size<br>Mainly male participants<br>Only from two countries<br>Not randomly recruited<br>Limited generalizability<br>Partial randomisation (*n*=3)<br>No comparison with placebo or another intervention<br>Loss to follow-up<br>Only 5 UAMs completed all three data collection points<br>CRIES-13 and CLS not culturally validated<br>Further follow-up needed
Rosner et al. (2020), Germany	RCT study protocol	TF-CBT/"Mein Weg"<br>Manualized<br>Group/Individual<br>Mein Weg: 9 group sessions<br>TF-CBT: 18 individual sessions	Trauma improvement	Collaboration with Child and Youth Welfare Services, private practices and outpatient clinics – Sessions will be conducted by therapists	540 UAMs<br>12-20 years old<br>PTSD	CATS-2, PHQ-9<br>GAD-7, AUDIT<br>WHOQOL-BREF<br>EQ-5D-3L, CYWS<br>GCIC, BAOS, SSQ<br>DSSYR, CUDIT-R<br>BSAS, HoNOSCA<br>CAMHSRI-REF		Possible recruitment difficulties<br>Strict inclusion criteria<br>Conflicting data on cultural validity of CATS<br>SSQ not culturally validated
Sarkadi et al. (2020), Sweden	RCT study protocol	TF-CBT<br>Teaching recovery techniques<br>Manualized<br>Group<br>5 weekly youth sessions, 2 caregiver sessions	Trauma improvement	Collaboration with community services – Sessions will be conducted by TRT-trained "group leaders"	218 UAMs<br>TRT: 109 UAMs<br>Control: 109 UAMs<br>14-20 years old<br>PTSD symptoms	CRIES-13, PHQ-9<br>GAD-7, GSE<br>CLS, Demographics questionnaire<br>RTHC, CHU-9D<br>TiC-P, C-SSRS		Possible recruitment difficulties<br>Strict inclusion criteria<br>Only self-report measures<br>CRIES-13 and CLS not culturally validated
Unterhitzenberger et al. (2019), Germany	Uncontrolled pilot study	TF-CBT<br>Manualized<br>Individual<br>15 weekly sessions	Trauma improvement	University Psychotherapy Outpatient Clinic<br>Sessions conducted by therapists	26 UAMs (M)<br>19 attended all sessions<br>15-19 years old<br>Afghanistan<br>PTSD, depression, behavioural and somatic symptoms<br>Accompanied by a	CATS<br>Kinder-DIPS<br>MFQ<br>SDQ<br>PHQ-15	Significant decrease in self-reported PTSS**, caregiver-reported PTSD***, depressive symptoms***, behavioural problems** and physical complaints*	Small sample size<br>Only male participants<br>Approx. 75% from one country<br>Strict inclusion criteria<br>Not randomly recruited<br>Limited generalizability<br>No control group<br>No comparison with placebo or

					caregiver/social worker			another intervention<br>Conflicting data on cultural validity of CATS and SDQ<br>Kinder-DIPS not culturally validated<br>Language barriers<br>Loss to follow-up
Van Es et al. (2021), Netherlands	Uncontrolled study	Multimodal approach:<br>TF-CBT, KidNET, EMDR<br>Individual<br>4-17 sessions	Trauma improvement	Collaboration with the national guardianship institution for UAMs and separated children<br>Sessions conducted by therapists	41 UAMs (14F)<br>17 attended all sessions<br>12-19 years old<br>Eritrea, Syria, Afghanistan<br>PTSD, depression	CRIES-13, PHQ-A<br>Demographics<br>Request for help<br>Life events (KidNET lifeline)<br>Programme integrity (exposure and adherence calculation)<br>Feasibility questionnaire	Adjustments made to treatment protocol; factors limiting the approach feasibility were identified; significant decrease in PTSD symptoms (T1 to T3***); non-significant change in depression symptoms	Small sample size<br>Mainly male participants<br>Approx. 75% from one country<br>Not randomly recruited<br>Limited generalizability<br>No control group<br>No comparison with placebo or another intervention<br>No manual<br>CRIES-13 not culturally validated<br>Language barriers<br>Missing data<br>Many sessions did not contain the planned treatment-Difficulty in impact assessment<br>No independent intervention assessment<br>Selective recruitment of therapists<br>No follow-up
Walg et al. (2020), Germany	RCT	Multimodal approach:<br>CBT, DBT, TPT Stabilisation Training for Trauma Induced Disorders<br>Group<br>6 weekly sessions	Trauma improvement	Inpatient Youth Welfare Facilities<br>Sessions conducted by two educational staff members	49 UAMs (M)<br>Intervention: n=23<br>Control: n=26<br>15-18 years old<br>Mainly from Afghanistan and Syria<br>Mental distress	BSCL<br>CBCL6-18/R	Reduction of self-reported mental distress in intervention group**<br>Reduction of externally assessed mental distress in both groups*	Small sample size<br>Only male participants<br>Approx. 50% from one country<br>Strict inclusion criteria<br>Not randomly recruited<br>Limited generalizability<br>Attendance rates varied<br>No comparison with placebo or another intervention<br>No manual<br>BSCL not culturally validated<br>Further follow-up needed
		**Art-based interventions**						
DeMott et al. (2017), Norway	Controlled study	Expressive Arts In Transition (EXIT)<br>Manualized<br>Group<br>10 sessions (2 per week)	Trauma improvement<br>Promotion of wellbeing	Arrival Centre for UAMs and other refugee facilities – Sessions conducted by therapists	143 UAMs (M)<br>EXIT: 70 UAMs<br>LAU: 73 UAMs<br>15-18 years old<br>Afghanistan, Somalia, Iran, Western Sahara, Palestine, Algeria	SLE, HSCL-25<br>HTQ, CLS	-Post-traumatic symptoms: Significant time by group interaction*<br>-General distress: No significant findings<br>-Life satisfaction: Significant time by group interaction* and group differences at T2*, T4*** and T5**<br>EXIT- significant increases in time (T1 to T3*, T4***, T5*** and T2 to T4**, T5** and T3 to T4*, T5*)<br>Expected life satisfaction:	Only male participants<br>Mainly from two countries<br>Not randomly recruited<br>Limited generalizability<br>Partial randomisation<br>Loss to follow-up<br>Only self-report measures<br>SLE and CLS not culturally validated

							Significant group differences at T4* and T5**	
		**Systemic interventions**						
Ochoa de Alda Martinez de Apellaniz et al. (2009), Spain	Uncontrolled pilot study	Systemic<br>Group<br>10 sessions	Mental health improvement	Collaboration with NGO that manages foster homes<br>Sessions conducted by two Family Therapists	12 UAMs (M)<br>12-14 years old<br>Morocco<br>Depression, alexithymia, low self-esteem	CDS, RSE, TAS-20<br>Ad hoc questionnaire (satisfaction with family relationships)	Significant decrease in alexithymia and depression scores***<br>Significant increase in self-esteem***	Small sample size<br>All participants from the same country<br>Not randomly recruited<br>Limited generalizability<br>Only self-report measures<br>No PTSD measures<br>No control group<br>No comparison with placebo or another intervention<br>Effects not demonstrated by two different investigators<br>No follow-up
	<u>Mixed-method studies</u>							
		**CBT-based interventions**						
Sarkadi et al. (2018), Sweden	Uncontrolled study	TF-CBT<br>Teaching recovery techniques<br>Manualized<br>Group<br>5 weekly youth sessions, 2 caregiver sessions	Trauma improvement	Collaboration with community and health services<br>Sessions conducted by trained members of the staff	46 UAMs (3F)<br>10 groups<br>14-18 years old<br>Mainly Afghanistan & Syria<br>PTSD	CRIES-8, MADR-S<br>Interviews	Significant reduction in PTSD* and depression symptoms***<br>URMs with suicidal ideation were more likely to report reduced depression symptoms*<br>22% UAMs were classified as recovered on PTSD symptoms and 33% on depression symptoms<br><u>Qual. data</u>: Supported by the group, normalisation of experiences, valuable tools for daily life, making sense of what happened, managing feelings/having control, finding meaning/looking to the future	Small sample size<br>Mainly male participants from two countries<br>Not randomly recruited<br>Limited generalizability<br>No control group<br>No comparison with placebo or another treatment<br>Only self-report measures, possible effects of educational level<br>CRIES-8 and MADR-S not culturally validated
Scheiber et al. (2019), Austria	RCT pilot study	CBT<br>Preventive resilience training<br>Group<br>6 sessions	Mental health improvement	Refugee camp<br>Sessions conducted by mental health professionals trained in trauma therapy	55 UAMs (M)<br><u>Intervention</u>: n=18 (n=17 randomised)<br><u>Control</u>: n=37<br>14-17 years old<br>Afghanistan, Pakistan<br>Two interpreters<br>Caregivers	PROTECT Questionnaire<br>RHS-15<br>Several single-item questions, including general well-being<br>Interviews	Improvement of general wellbeing*<br>No significant decrease in trauma-related symptoms<br><u>Qual. data</u>: Psychoeducation, group activities and mutual support as sources of relief	Small sample size<br>Only male participants<br>Mainly from one country<br>Not randomly recruited<br>Limited generalizability<br>Partial randomisation<br>No comparison with placebo or another intervention<br>Only self-report measures<br>No culturally validated measures<br>No follow-up
Van der Gucht et al. (2019),	Uncontrolled pilot study	Mindfulness<br>Manualized	Mental health improvement	Shelter for UAMs<br>Sessions conducted by	13 UAMs (5F)<br>9 attended all sessions	I-PANAS-SF<br>CRIES	Significant decrease in negative affect* and	Small sample size<br>Not randomly recruited

Belgium		Group<br>8 weekly sessions		two Mindfulness trainers	3 groups<br>13-18 years old<br>Afghanistan, Albania, Syria, Russia, Bosnia, Angola, Pakistan, Rwanda, Somalia<br>No current severe mental illness	Semi-structured interviews	symptoms of depression*<br>Significant increase in positive affect*<br>Qual. data:<br>*Expectations*: reducing stress and trauma-related symptoms and social integration<br>*Experiences*: concerns about the format, duration and language of the program<br>*Integration*: mindfulness exercises used as a new coping strategy in combination with familiar coping strategies	Limited generalizability<br>No control group<br>No comparison with placebo or another intervention<br>Only self-report measures<br>Conflicting data on cultural validity of I-PANAS-SF<br>CRIES not culturally validated<br>Organisational and practical issues<br>High attrition rate<br>No further follow-up
		**Narrative Exposure Therapy**						
Said & King (2020), UK	Uncontrolled pilot study	NET<br>Individual<br>9-20 sessions	Trauma improvement	NHS, CAMHS for people from refugee backgrounds<br>Sessions conducted by final-year clinical psychology doctoral trainees under supervision	4 UAMs (1F)<br>3 attended all sessions<br>16-17 years old<br>Sudan, Vietnam, Albania<br>Suicidal ideation, self-harming, PTSD	CRIES-8, CPSS-5<br>Semi-structured interviews	2 UAMs' symptom scores were below the clinical range for PTSD post-treatment<br>3 UAMs showed reliable improvement<br>2 no longer engaged in self-harming behaviour<br>None reported suicidal ideation post-treatment<br>Qual. data: Improved concentration, fewer intrusive memories, less distress, increased cohesion and sense-making	Small sample size<br>Mainly male participants<br>Not randomly recruited<br>Limited generalizability<br>No control group<br>No comparison with placebo or another intervention<br>Possible effects of other interventions implemented alongside NET<br>Only self-report measures<br>Only PTSD measures<br>No culturally validated measures<br>Incomplete outcome data for one participant<br>No follow-up
		**Other interventions**						
Garoff et al. (2019), Finland	Uncontrolled pilot study	Multimodal approach:<br>Phase-based approach of trauma care, IASC Guidelines for MHPSS<br>Manualized<br>Individual/Group<br>10 weekly group sessions and individual sessions	Trauma improvement<br>Mental health improvement<br>Professionals' training	Accommodation facilities<br>Implementation by mental health professionals and staff members, consultation by the research team	18 UAMs (2F)<br>Group: n=17<br>Individual: n=1<br>9-17 years old<br>Mainly Afghanistan and Iraq<br>PTSD symptoms<br>9 staff members<br>2 mental health professionals	Semi-structured interviews<br>Focus Group Discussions<br>Brief ethnographic interviewing<br>Resilience self-rated scale<br>CRIES, SDQ	No significant changes in mental health symptoms<br>Qual. data: Increased trust and communication, empowerment for group facilitation	Difficulties in recruitment and group formation<br>Small sample size<br>Mainly male participants from two countries<br>Not randomly recruited<br>Limited generalizability<br>No control group<br>No comparison with placebo or another intervention<br>Possible effects of ongoing medication in some participants<br>Only self-report measures<br>Conflicting data on cultural validity of SDQ<br>CRIES not culturally validated<br>Difficulties in the completion of the self-rated questionnaires in a group setting (comprehension difficulties and social anxiety)

								Talking about difficult issues in a group setting not appealing for some participants<br>Variations in understanding and responding to interview questions were not systematically recorded<br>No follow-up
<u>Qualitative studies</u>								
		**CBT-based interventions**						
Unterhitzenberger et al. (2021), Germany	Content analysis	TF-CBT<br>Manualized Training and supervision for therapists working with UAMs in a pilot study – see Unterhitzenberger et al. (2019)<br>Individual/Group	Professionals' training<br>Improvement of the therapy	University Psychotherapy Outpatient Clinic<br>TF-CBT training and biweekly supervision by a certified trainer<br>Monthly case consultation by manual developers<br>Half-day training session by an expert in psychotherapy with refugees and torture survivors	9 therapists (8F)<br>29-60 years old	Session checklist for the TF-CBT modules<br>Survey for each case after completion	Adjustments made to treatment protocol, few obstacles identified, the manual was considered helpful	Small sample size<br>Not randomly recruited<br>Limited generalizability<br>No control group<br>No comparison with placebo or another intervention<br>Possible bias in question phrasing<br>No calculation of interrater reliability scores<br>Lack of objective ratings<br>No previous assessment of the therapists' cultural competence<br>No assessment of UAMs' feedback<br>No follow-up
		**Narrative Exposure Therapy**						
Said et al. (2021), UK	IPA<br>Further analysis of Said & King's (2020) study	NET<br>Individual<br>9-20 sessions	Trauma improvement	NHS, CAMHS for people from refugee backgrounds<br>Interviews conducted by a master's student	4 UAMs (1F)<br>16-17 years old<br>Sudan, Vietnam, Albania<br>PTSD	CRIES-8, CPSS-5<br>Interviews	Understanding PTSD, integrating trauma in life narrative, increased self-efficacy and expression, decreased symptomatology, post-traumatic growth	Small sample size<br>Mainly male participants<br>Not randomly recruited<br>Limited generalizability<br>No control group<br>No comparison with placebo or another intervention<br>Only self-report measures<br>No culturally validated measures<br>Language barriers<br>Participants were at different stages of their treatment when interviews were conducted<br>Participants knew of their therapist's involvement in the study
		**Transcultural interventions**						
Radjack et al. (2020), France	Thematic analysis<br>Narrative and transcultural approaches	A participative action research programme to transmit cultural competence to social workers who provide care for UAMs<br>Individual	Professionals' training<br>Trauma improvement	University hospital<br>Psychiatric department for children and adolescents<br>Meetings were conducted by mental health professionals	29 UAM (3F)<br>16-18 years old<br>Sub-Saharan Africa, North Africa, and Southern Asia<br>PTSD, depression, addictions, somatic complaints (*n*=8)<br>29 Social Workers	Semi-structured interviews (2 to 3 interviews per dyad)<br>Three tools (objects, the circle test, and cultural mediators)	Understanding the experience of being a UAM (*A suspended future/ religion as guarantee of continuity/ preserving one's dignity/ difficulty of escaping a sense of loneliness*)<br>Promoting changes in the youth and in the	Small sample size<br>Mainly male participants<br>Not randomly recruited<br>Limited generalizability<br>No control group<br>No comparison with placebo or another intervention<br>No standardised measures of mental health and progress

		3 monthly meetings					relationship between the youth and the social worker (*Taking into account the experiences before, during, and after migration/ from "I" to "we"/ a mediator that facilitates bridges between the cultural worlds*)	8 UAMs who were lost to follow-up and had only a single interview are included in the analysis
Radjack et al. (2021), France	Thematic analysis<br>Further analysis of Radjack et al. (2020) study	A participative action research programme to transmit cultural competence to social workers who provide care for UAMs<br>Individual<br>3 monthly meetings	Professionals' training<br>Trauma improvement	University hospital<br>Psychiatric department for children and adolescents<br>Meetings were conducted by mental health professionals	17 UAMs (3F)<br>15 attended all sessions<br>17-18 years old<br>Sub-Saharan Africa, North Africa, and Southern Asia<br>PTSD, depression, addictions, somatic complaints (*n*=7)<br>17 Social Workers<br>Interpreters-mediators	Semi-structured interviews (2 to 3 interviews per dyad)<br>Three tools (objects, the circle test, and cultural mediators)	Enhancement of professionals' cultural competence<br>Re-activation of UAMs' identity construction<br>*(Effective sub-themes for an action modifying the relationship between the young person and the social worker: need to get rid of a sense of helplessness; highlight the minor's ability to connect and not being afraid belonging to a group; give meaning to the journey, such as a rite of passage; assume a maternal position for the social worker)*	Small sample size<br>Mainly male participants<br>Not randomly recruited<br>Limited generalizability<br>No control group<br>No comparison with placebo or another intervention<br>No standardised measures of mental health and progress<br>Loss to follow-up
<u>Case studies/series</u>								
		**CBT-based interventions**						
Bræin et al. (2011), Norway	Case series	Multimodal approach:<br>Phase-oriented treatment model, TF-CBT, NET/Kid-NET<br>Individual<br>1 year	Trauma improvement	Outpatient clinic<br>Sessions conducted by psychologists	2 UAMs (M)<br>Afghanistan, Middle East<br>PTSD, Developmental Trauma Disorder, Complicated Grief Disorder	Leiter-R	Fewer intrusive thoughts, partial improvement in social relationships and academic performance, not fulfilling criteria for diagnosis	Small sample size<br>Only male participants<br>No age indicated<br>Not randomly recruited<br>Limited generalizability<br>No control group<br>No comparison with placebo or another intervention<br>No standardised measures of progress<br>No manual<br>Effects not demonstrated by different investigators<br>No follow-up
Franco (2018), USA	Case study<br>Part of literature review	TF-CBT<br>School-based intervention<br>Individual/Group<br>10-month weekly group meetings, individual	Trauma improvement	Public high school<br>Sessions conducted by a social worker	1 UAM (F)<br>16 years old<br>Central America<br>Sadness, trouble sleeping, flashbacks of traumatic event, weight loss		Improved class effort and school attendance)	Results not generalizable because of single case study<br>Not randomly recruited<br>No control group<br>No comparison with placebo or another intervention<br>No standardised measures of

		sessions, sessions<br>with the caregiver						mental health and progress<br>Effects not demonstrated by<br>different investigators<br>No follow-up
King & Said<br>(2019), UK	Case study	Multimodal<br>approach:<br>CBT<br>TF-CBT<br>STAIR<br>CFT<br>Group<br>35 weeks	Mental health<br>improvement<br>Promotion of<br>wellbeing	NHS, CAMHS for<br>refugees and asylum<br>seekers<br>Sessions conducted by<br>psychologists	14 UAMs (1F)<br>14-17 years old<br>Afghanistan, Ethiopia,<br>Sudan, Somalia	Attendance record<br>CGSRS, RCI<br>SDQ, CRIES-8<br>Open discussions for<br>feedback	4 UAMs showed a reliable<br>improvement in emotional<br>and behavioural wellbeing,<br>but not in risk of self-harm<br>Positive feedback about<br>being part of the group,<br>learning, feeling welcome,<br>respected and less stressed	Small sample size<br>Mainly male participants<br>Not randomly recruited<br>Limited generalizability<br>No control group<br>No comparison with placebo or<br>another intervention<br>Missing data<br>Attendance rates varied<br>Only self-report measures<br>Conflicting data on cultural validity<br>of SDQ<br>CRIES-8 not culturally validated<br>Effects not demonstrated by<br>different investigators<br>No follow-up
Unterhitzenberg<br>er et al. (2015),<br>Germany	Case series	TF-CBT<br>Manualized<br>Individual<br>12-28 sessions	Trauma<br>improvement	Trauma Outpatient<br>Clinics<br>Sessions conducted by<br>therapists	6 UAMs (2F)<br>16-18 years old<br>Somalia, Afghanistan,<br>Iran<br>PTSD	CAPS-CA, PDS	Significant decrease of PTSD<br>symptoms***	Small sample size<br>Not randomly recruited<br>Limited generalizability<br>No control group<br>No comparison with placebo or<br>another intervention<br>No culturally validated measures<br>Differences in number of sessions,<br>involvement of caregivers and<br>assessment<br>No follow-up
Unterhitzenberg<br>er & Rosner<br>(2016),<br>Germany	Case study	TF-CBT<br>Manualized<br>Individual<br>12 youth sessions<br>12 caregiver<br>sessions	Trauma<br>improvement	Outpatient setting<br>Sessions conducted by<br>a therapist	1 UAM (F)<br>17 years old<br>East Africa<br>PTSD, symptoms of<br>depression and anxiety	CAPS-CA<br>UCLA-PTSD<br>CDI, SCARED	Recovery from PTSD,<br>decrease in anxiety and<br>depression symptoms	Results not generalizable<br>because of single case<br>study<br>Not randomly recruited<br>Conflicting data on cultural validity<br>of CDI and SCARED<br>UCLA-PTSD not culturally<br>validated<br>Effects not demonstrated<br>by different investigators
Unterhitzenberg<br>er et al. (2019),<br>Germany	Case study	TF-CBT<br>Manualized<br>Individual<br>15 sessions	Trauma<br>improvement	Outpatient setting<br>Sessions conducted by<br>a therapist	1 UAM (M)<br>17 years old<br>Afghanistan<br>PTSD, major depression	CATS	Recovery from PTSD	Results not generalizable because<br>of single case study<br>Not randomly recruited<br>Only self-report measures<br>Conflicting data on cultural validity<br>of CATS<br>Effects not demonstrated by<br>different investigators<br>No follow-up
Vickers (2005),<br>UK	Case study	CBT<br>Individual<br>16 sessions	Trauma<br>improvement	CAMHS<br>Sessions conducted by<br>a therapist	1 UAM (F)<br>14 years old<br>Africa<br>Severe PTSD, mild	PDS	Decrease in PTSD symptoms	Results not generalizable<br>because of single case<br>study<br>Not randomly recruited

					depression			Only self-report measures<br>No culturally validated measures<br>Effects not demonstrated by different investigators<br>No follow-up
		**Psychodynamic-based interventions**						
Cohen & Yadlin (2018), UK	Case series	Psychodynamic<br>Individual	Trauma improvement	CAMHS<br>Sessions conducted by child psychotherapists	3 UAMs (1F)<br>14-16 years old<br>Episodes of confusion and physical collapse, low mood, recurrent nightmares, excessive sleep and suicidal thoughts, dissociation		Sharing feelings more openly, recovering positive sense of self, although feelings of distress and trauma may have remained	Small sample size<br>No country of origin indicated<br>Not randomly recruited<br>Limited generalizability<br>No control group<br>No comparison with placebo or another intervention<br>No standardised measures for mental health and progress<br>No manual<br>No indication of therapy duration<br>No follow-up
Katsounari (2014), USA	Case study	Multimodal approach:<br>Relational psychodynamic, TF-CBT<br>Individual<br>16 sessions	Trauma improvement	Locked facility for children who have entered the United States without their parents or documentation<br>Sessions conducted by a therapist	1 UAM (M)<br>16 years old<br>Latino<br>PTSD with dissociative features, major depression	Semi-structured diagnostic biopsychosocial intake interview	Decrease of PTSD symptoms, nightmares, flashbacks, dissociative episodes, feeling safer, better relationships	Results not generalizable because of single case study<br>Not randomly recruited<br>No standardised measures of mental health and progress<br>No manual<br>Effects not demonstrated by different investigators<br>No follow-up
Melzak (2009), UK	Case study	Multimodal approach:<br>Psychoanalytic - Developmental<br>Individual<br>3 Years	Trauma improvement	The Baobab Centre (non-residential, transitional therapeutic community for children, adolescents and young adults)<br>Sessions conducted by child psychotherapists	1 young adult- former UAM (M)<br>Africa<br>Sadness, intrusive thoughts, nightmares, suicidal ideas			Results not generalizable because of single case study<br>No indication of age<br>Not randomly recruited<br>No standardised measures of mental health and progress<br>No manual<br>Effects not demonstrated by different investigators<br>No indication of effects of the intervention on mental health<br>No follow-up
Melzak. (2017), UK	Case study	Multimodal approach:<br>Psychoanalytic - Developmental<br>Individual	Trauma improvement<br>Promotion of wellbeing	The Baobab Centre<br>Sessions conducted by child psychotherapists	1 young adult- former UAM (M)<br>21 years old<br>China<br>Trauma		Recalling repressed feelings of anger after sharing experiences	Results not generalizable because of single case study<br>Not randomly recruited<br>No standardised measures of mental health and progress<br>No indication of intervention duration<br>No manual<br>Effects not demonstrated by different investigators<br>No follow-up
		**Narrative-based interventions**						

Draper et al. (2022), UK	Case series	Narrative/social constructionist approach<br>"Continuing Bonds" method<br>Individual	Mental health improvement	NHS<br>Sessions conducted by therapists	2 UAMs (M)<br>16/19 years old<br>Anxiety, loss of interest, difficulties in school attendance, migratory grief	Session transcripts	Safety and connectedness through discussing identity, hope, bonds, and time	Small sample size<br>Only male participants<br>Not randomly recruited<br>No country of origin indicated<br>Limited generalizability<br>No control group<br>No comparison with placebo or another intervention<br>No standardised measures for mental health and progress<br>No manual<br>No follow-up
Jacobs (2017), Greece	Case study	"The Tree of Life"<br>Group<br>2 sessions	Mental health improvement<br>Promotion of wellbeing	Shelter of PRAKSIS NGO<br>Sessions conducted by a Family Therapist	8 UAMs (M)<br>Bangladesh, Pakistan, Afghanistan, Syria, Ghana, Iraq		Connectedness, transformation in how they perceived their problems and hardships	Small sample size<br>Only male participants<br>No age indicated<br>Not randomly recruited<br>Limited generalizability<br>No control group<br>No comparison with placebo or another intervention<br>No standardised measures of mental health and progress<br>Effects not demonstrated by different investigators<br>No follow-up
		**Transcultural interventions**						
Benoit de Coignac & Baubet (2013), France	Case study	Transcultural<br>Individual<br>Monthly meetings	Trauma improvement	University hospital<br>Psychiatric department for children and adolescents	1 UAM (M)<br>17 years old<br>Ivory Coast<br>Mental suffering, possession state		Feeling good during meetings, due to therapists' responses	Results not generalizable because of single case study<br>Not randomly recruited<br>No standardised measures of mental health and progress<br>No manual<br>No indication of intervention duration<br>Effects not demonstrated by different investigators<br>No follow-up
Bouaziz et al. (2013), France	Case study	Transcultural<br>Individual<br>Monthly meetings	Mental health improvement	University hospital<br>Multidisciplinary group of therapists (psychiatrists, psychologists, nurses, anthropologists)	1 UAM (M)<br>Adolescent<br>Bangladesh<br>Psychotic symptoms, complex PTSD, depression			Results not generalizable because of single case study<br>Not randomly recruited<br>No standardised measures of mental health and progress<br>No indication of intervention duration<br>No manual<br>Effects not demonstrated by different investigators<br>No follow-up
Radjack et al. (2012), France	Case study	Transcultural<br>Individual	Trauma improvement	University hospital<br>Group of therapists	1 UAM (M)<br>17 years old<br>Afghanistan<br>Sadness, withdrawal, distrust		Improvement of self-esteem and confidence	Results not generalizable because of single case study<br>Not randomly recruited<br>No standardised measures of mental health and progress

								No manual<br>No indication of intervention duration<br>Effects not demonstrated by different investigators<br>No follow-up
Touhami et al. (2015), France	Case study	Transcultural<br>Individual<br>3 sessions	Mental health improvement	University hospital<br>Psychiatric department for children and adolescents<br>Multidisciplinary group of therapists	1 UAM (M)<br>17 years old<br>Senegal<br>Withdrawal<br>Accompanied by educator		Increased sense of belonging and possibility of educational re-evaluation	Results not generalizable because of single case study<br>Not randomly recruited<br>No standardised measures of mental health and progress<br>No manual<br>Effects not demonstrated by different investigators<br>No follow-up
		**Other interventions**						
Alfano et al. (2017), Italy, Switzerland, France	Case study	Photolanguage<br>Group<br>10 weekly sessions	Mental health improvement	Part of a care and psychological support project for UAMs<br>Sessions conducted by a clinical psychologist	5 UAMs (M)<br>15-17 years old<br>Sub-Saharan Africa, Bangladesh<br>Trauma	Photographs - discussion	Enabled transformation and change	Small sample size<br>Not randomly recruited<br>Limited generalizability<br>No control group<br>No comparison with placebo or another intervention<br>No standardized measures of mental health and progress<br>No manual<br>Effects not demonstrated by different investigators<br>No follow-up
Mattenschlager et al. (2016), Germany	Case study	Psychodrama<br>Individual/Group	Mental health improvement<br>Promotion of wellbeing	Psychological Family- and Life- Counselling Service of Caritas Ulm<br>Sessions conducted by psychotherapists	Individual: 24 children and young people (2/3 UAMs)<br>Syria, Eritrea, Somalia, Iran, Afghanistan, North Macedonia, Bulgaria<br>Group: 4 teenagers (1F)<br>Eritrea<br>Trauma		Improved mental health and development, if combined with stable external conditions	Small sample size<br>Mainly male participants<br>Not randomly recruited<br>Limited generalizability<br>No control group<br>No comparison with placebo or another intervention<br>No standardised measures of mental health and progress<br>No manual<br>Effects not demonstrated by different investigators<br>No follow-up
Mehraby (2002), Australia	Case study	Multimodal approach:<br>Model of trauma and recovery<br>Group<br>5 sessions	Trauma improvement	NSW Service for the Treatment and Rehabilitation of Torture and Trauma Survivors<br>Sessions conducted by counsellors	14 UAMs (3F)<br>12-21 years old<br>Afghanistan<br>Severely traumatized		Creation of safe environment for sharing and normalizing experiences, facilitating empowerment	Small sample size<br>Mainly male participants<br>Only from one country<br>Not randomly recruited<br>Limited generalizability<br>No standardised measures of mental health and progress<br>No manual<br>Effects not demonstrated by different investigators<br>No indication of effects of the intervention on mental health

							No follow-up
Practice papers							
	**CBT-based interventions**						
Alto et al. (2021), USA	Multimodal approach:<br>IPT-A, TF-CBT, CBT<br>Individual/Group<br>9 group sessions and individual sessions	Mental health improvement<br>Professionals' training	Congress funded programme run by a MHO for children and families at risk<br>Collaboration with a foster care program, the Department of Human Services case managers, the Expert Agency, and the local school program for newcomer youth	30 UAMs (F/M)<br>Individual: 13 UAMs<br>Group: 17 UAMs (6F)<br>15-20 years old<br>Guatemala, Honduras, Mexico, Eritrea, Nepal<br>Trauma, depression<br>Over 90 community partners serving refugees<br>Foster parents of UAMs	Feedback Form	Positive feedback - connection, support, sharing, safety, care, learning helpful strategies	Small sample size<br>Not randomly recruited<br>Limited generalizability<br>No research design<br>No control group<br>No comparison with placebo or another intervention<br>No standardised measures of mental health and progress<br>No follow-up
Simons & Pöter (2017), Germany	Multimodal approach:<br>Metacognitive therapy<br>Pharmacotherapy<br>Individual	Mental health improvement	Refugee Outpatient Clinic<br>Sessions conducted by psychiatrists	UAMs<br>Mainly male<br>12-17 years old<br>Afghanistan, Syria, various African countries<br>PTSD		The intervention seems helpful with this target group	Undefined number of participants<br>Not randomly recruited<br>Limited generalizability<br>No research design<br>No control group<br>No comparison with placebo or another treatment<br>No standardised measures of mental health and progress<br>No follow-up
	**Psychodynamic-based interventions**						
Melzak (2018), UK	Multimodal approach:<br>Psychoanalytic-Developmental, ADAPT<br>daily activities, community meetings<br>Individual/Group	Promotion of wellbeing	The Baobab Centre<br>Sessions conducted by Child psychotherapists	5 UAMs (1F)<br>16-23 years old<br>Middle East, Africa<br>Trauma		Possible alleviation of extreme protective defence mechanisms<br>Hope of psychological survival and emotional recovery	Not randomly recruited<br>Limited generalizability<br>No research design<br>No control group<br>No comparison with placebo or another intervention<br>No standardised measures of mental health and progress<br>No manual<br>No indication of intervention duration<br>No follow-up
	**Art-based interventions**						
DeMott (2017), Norway	Expressive Arts in Transition (EXIT)<br>Group<br>10 twice-weekly sessions	Trauma improvement<br>Promotion of wellbeing	Arrival Centre for UAMs<br>Sessions conducted by a therapist	71 UAMs (M)<br>15-18 years old<br>Mainly Middle East and Africa<br>40% of them PTSD		Reduction in posttraumatic stress, anxiety and depression<br>Quality of life and optimism about the future increased	Only male participants<br>Not randomly recruited<br>Limited generalizability<br>No research design<br>No control group<br>No comparison with placebo or another intervention<br>No standardised measures of mental health and progress<br>Effects not demonstrated by different investigators

							No follow-up
	**Other interventions**						
Neurohr & Willner (2019), Germany	Multimodal approach: Psychotherapeutic crisis intervention, Ego-supportive therapeutic approach<br>Individual	Mental health improvement	Initial Reception Centre<br>Sessions conducted by an external psychotherapist	2 UAMs (M)<br>16-17 years old<br>Afghanistan<br>Post-traumatic and depressive symptoms	CASCAP-D<br>ICD-10 criteria	Reduction of post-traumatic and depressive symptoms, suicidal tendencies, auto-aggressive behaviour, and substance abuse; strengthened ego skills	Small sample size<br>Only male participants from one country<br>Not randomly recruited<br>Limited generalizability<br>No research design<br>No control group<br>No comparison with placebo or another intervention<br>No culturally validated measures<br>No indication of intervention duration<br>Effects not demonstrated by different investigators<br>No follow-up

Note: **ADAPT** = Adaption and Development After Persecution and Trauma model, **AM** = Accompanied Minor, **AUDIT** = Alcohol Use Disorder Identification Test, **BAOS** = Brief Acculturation Orientation Scale, **BSCL** = Brief Symptom Checklist, **BSAS** = Brief Sociocultural Adaptation Scale, **CAMHSRI-REF** = Child and Adolescent Mental Health Service Receipt Inventory adapted for refugee youths, **CAMHS** = Child and Adolescent Mental Health Service, **CAPS-CA** = Clinician Administered PTSD Scale for Children and Adolescents, **CASCAP-D** = Children's Anger Control and Social Problem-Solving Scale, **CATS** = Child and Adolescent Trauma Screen, **CATS-2** = Child and Adolescent Trauma Screen, second version, **CATS-Care** = Child and Adolescent Trauma Screen (caregiver report), **CATS-Self** = Child and Adolescent Trauma Screen (self-report), **CBCL/6-18** = Child Behavior Checklist for Ages 6-18, **CBT** = Cognitive Behavioral Therapy, **CDI** = Children's Depression Inventory, **CDS** = Child Depression Scale, **CFT** = Compassion Focused Therapy, **CGSRS** = Child Group Session Rating Scales, **CHU-9D** = Child Health Utility Instrument, **CLS** = Cantril's Ladder of Life Satisfaction, **CPSS-5** = Child PTSD Symptom Scale for DSM-5, **CPTCI-S** = Child Post-Traumatic Cognitions Inventory, **CRIES** = Child Revised Impact of Events Scale, **CRIES-13** = Child Revised Impact of Events Scale – 13, **C-SSRS** = Columbia-Suicide Severity Rating Scale, **CUDIT-R** = Cannabis Use Disorder Identification Test Revised, **CYWS** = Child and youth welfare service, **DBT** = Dialectical Behavioural Therapy, **DSRS-C** = Birleson Depression Self-Rating Scale for Children, **DSSYR** = Daily Stressors Scale for Young Refugees, **EMAG** = Ethnic Minority Achievement Group, **EQ-5D-3L** = Quality of Life Assessment Scale, **EXIT** = Expressive Arts in Transition, **F** = Female, **GAD-7** = Generalized Anxiety Disorder 7, **GCIC** = Group Climate Instrument for Children, **GSE** = General Self-Efficacy Scale, **HoNOSCA** = Health of the Nation Outcome Scales for Children and Adolescents, **HSCL-25** = Hopkins Symptom Checklist-25, **HTQ** = Harvard Trauma Questionnaire, **IASC** = Inter-Agency Standing Committee, **ICD-10** = International Classification of Diseases 10th revision, **IPA** = Interpretative phenomenological analysis, **I-PANAS-SF** = International Positive and Negative Affect Schedule – Short Form, **IPT-A** = Interpersonal Psychotherapy for Adolescents, **Kid-NET** = Narrative Exposure Therapy for Children, **Kinder-DIPS** = Diagnostic Interview for Mental Disorders in Children and Adolescents, **LAO** = Life As Usual, **Leiter-R** = Leiter International Performance Scale – Revised, **M** = Male, **MADR-S** = Montgomery-Asberg Depression Rating Scale, **MHO** = Mental Health Organisation, **MHPSS** = Mental Health Psychosocial Support, **NET** = Narrative Exposure Therapy, **NHS** = National Health System, **PDS** = Posttraumatic Diagnostic Scale, **PHQ-8** = Eight-item Patient Health Questionnaire depression scale, **PHQ-9** = Patient Health Questionnaire-9, **PHQ-A** = The Patient Health Questionnaire-9 modified for Adolescents, **PROTECT Questionnaire** = Detection of mental health problems in asylum seekers by non-health professionals, **PTSD** = Post-Traumatic Stress Disorder, **PTSS** = Post-Traumatic Stress Syndrome, **RCMAS** = Revised Children's Manifest Anxiety Scale, **RCT =** Randomised Controlled Trial, **RHS-15** = Refugee Health Screener - 15, **R-IES** = Revised Impact of Event Scale, **RSES** = Rosenberg Self-Esteem Scale, **RTHC** = Refugee Trauma History Checklist, **SCARED** = Screen for Child Anxiety Related Disorders, **SDQ** = Strengths and Difficulties Questionnaire, **SLE** = Stressful Life Events Checklist, **SSQ** = Social Support Questionnaire, **STAIR** = Skills Training in Affective and Interpersonal Regulation, **TAS-20** = Toronto Alexythimia Scale, **TF-CBT** = Trauma-Focused Cognitive Behavioural Therapy, **TiC-P** = Treatment Inventory of Costs in Psychiatric Patients, **TPT** = Time Perspective Therapy, **TRT** = Teaching Recovery Techniques, **UAM** = Unaccompanied Minor, **UCLA-PTSD** = The University of California at Los Angeles Posttraumatic Stress Disorder Reaction Index for DSM-5, **WHOQOL-BREF** = World Health Organization Quality of Life, brief version, **WTQ** = War Trauma Questionnaire

* $p < 0.05$, ** $p < 0.01$, *** $p < 0.001$
