# Supplementary material for: Psychotherapeutic and Psychosocial Interventions with Unaccompanied Minors: A Scoping Review
Source: Healthcare (Basel). 2023 Mar 22;11(6):918. doi: 10.3390/healthcare11060918 (PMC10048295; doi:10.3390/healthcare11060918)
Supplement: Supplementary file 1 [file healthcare-11-00918-s001.zip › Table S4.pdf]

Table S4. *Overview of the 16 studies on PSS interventions*

**Author(s), Year, Country**	**Type of paper/study**	**Type of intervention**	**Aim of intervention**	**Setting**	**Participants**	**Measures (if any)**	**Results (if any)**	**Limitations (if any)**
Quantitative studies								
De Carolis et al. (2019), Italy	Uncontrolled experiment<br>Pilot study	Learning culture-related gestures with Social Robots<br>Individual	Integration	Laboratory setting<br>Intervention conducted by computer scientists	4 Italian children<br>6-10 years old<br>6 adult migrants<br>19-24 years old	A survey composed of 6 statements for evaluating the interaction with the Social Robot, the gesture recognition and execution	The proposed approach is suitable for learning gestures	Small sample size<br>Non-representative sample<br>Not randomly recruited<br>Limited generalizability<br>No control group<br>No comparison with placebo or another intervention<br>Effects not demonstrated by different investigators<br>No culturally validated measures<br>No baseline assessment<br>Only descriptive results<br>No follow-up
De Carolis et al. (2021), Italy	Uncontrolled experiment<br>Pilot study	Learning culture-related gestures with Social Robots<br>Comparison of new robot ("Nao") with the previous one ("Pepper") – see De Carolis et al., (2019)<br>Individual	Integration	Laboratory setting<br>Intervention conducted by computer scientists	10 Italian children<br>6-10 years old	A survey composed of 6 statements for evaluating the interaction with the Social Robot, the gesture recognition and execution	The proposed approach is suitable for learning gestures<br>The interaction with Pepper was better evaluated than the one with Nao for the learning gesture task***	Small sample size<br>Non-representative sample<br>Not randomly recruited<br>Limited generalizability<br>No control group<br>Comparison with an intervention of a previous study- Not matched samples<br>Effects not demonstrated by different investigators<br>No culturally validated measures<br>No follow-up
Mixed-method studies								
Alarcón et al. (2021), Spain	Controlled study	A mentoring programme for self-esteem, resilience and hope improvement<br>Individual<br>1 weekly meeting for 6 months	Wellbeing<br>Empowerment<br>Support	Punt de Referència Association, Barcelona metropolitan area<br>Mentoring conducted by trained adult volunteers	Quantitative:<br>44 UAMs (2F)<br>17-23 years old<br>Morocco, Algeria, Sub-Saharan countries, Latin America<br>*Mentoring*: n=21<br>*Control*: n=23<br>Qualitative:<br>10 mentees, their 10 mentors, and 10 non-mentored youths	RSE, CYRM-12<br>CHS, K10<br>Scale for educational aspirations<br>Semi-structured interviews	-**Self-esteem**: Significant increase in mentoring group*<br>-**Resilience**: Significant improvement in mentoring group from T1 to T2**<br>-**Youth hope**: Significant intervention × time interaction effect*, significantly stronger change in the mentoring group*<br>-**Psychological distress**: No significant findings<br>-**Educational**	Small sample size<br>Mainly male participants<br>Not randomly recruited<br>Limited generalizability<br>RSE and CYRM-12 not culturally validated<br>No manual<br>No significant intervention × time interaction effects for self-esteem and resilience<br>Possible effect of external factors<br>No follow-up

							**aspirations:** Significant improvement in mentoring group from T1 to T2** - **Educational expectations:** Significant improvement in mentoring group from T1 to T2** *Qualitative*: Mentoring as a means for emotional and social support, access to social capital, interest in formal educational paths	
Hidalgo et al. (2016), USA	Uncontrolled pilot study	"PATHS to Resilience" project - Enhancing the quality of relationships among staff and UAMs in residential care - addressing the clinical needs of traumatized youth<br>Group<br>2 ½day training	Improvement of caregivers' skills	Collaboration with four pilot sites<br>Intervention conducted by mental health professionals	297 staff members<br>55% YCWs<br>45% clinical or administrative residential facility staff- baseline data<br>160 attended the training	TABS, MHCI<br>Andrews & Withey Job Satisfaction Questionnaire<br>Administrative records<br>15 semi-structured in-depth interviews	YCWs reported statistically significant decrease in distress**<br>Staff perceived their residential facility to have a significantly higher level of mental health capacity*<br>Statistically significant improvement in self-reported job satisfaction*<br>Qualitative: 'Playfulness' as a means for communication's improvement and case management	Sample not randomly recruited<br>Limited generalizability<br>No control group<br>No comparison with placebo or another intervention<br>TABS not as sensitive as other measures to post-training effects<br>Unable to test the impact of each intervention component separately<br>Possible effect of funder on participants<br>No direct assessment of youth
	Qualitative studies							
Canizales (2015), USA	Ethnographic research	"Voces de Esperanza"<br>Group<br>1 two-hour meeting per week	Wellbeing<br>Empowerment<br>Support	Informal support group<br>Led by two coordinators	Group: 40 members 18-31 years old (former UAMs) Guatemala<br>Research participants: 2 group coordinators, 15 members (2F)	Researcher's notes from the group meetings<br>Semi-structured in-depth interviews	Adopt expressive individualism (personal narrative development and self-construction).<br>Shift social commitments (economic attachments, cultural identity, the self as role model)	Small sample size<br>Mainly male participants<br>Only from one country<br>Not randomly recruited<br>Limited generalizability<br>No control group<br>No comparison with placebo or another intervention<br>Effects not demonstrated by different investigators<br>No standardised measures of mental health and progress<br>No manual<br>No follow-up
Clacherty (2021), South Africa	Art-based, narrative research	"The suitcase project"<br>An art-based project for war-affected children	Wellbeing<br>Empowerment<br>Support	Initially an informal group run voluntarily by a child research	27 UAMs<br>(just over 1/3F)<br>6-18 years old	Transcripts of the group discussions and artwork	Meaning making<br>Self-expression through art	Small sample size<br>Only participants from African countries

		and young people who cross borders<br>Group		practitioner and an artist at a local school of Johannesburg	Rwanda, Burundi, DRC, Zimbabwe, Ethiopia, Angola		Apprehend their own strength and agency	Not randomly recruited<br>Limited generalizability<br>No control group<br>No comparison with placebo or another intervention<br>Effects not demonstrated by different investigators<br>No standardised measures of mental health and progress<br>No indication of intervention duration<br>No follow-up
Kakowa & Kaomba (2020), Malawi	Content and thematic analysis	Kinship care system for PSS<br>Various methods:<br>1. "Tumbo moja" (Bereavement councelling)<br>2. "Watoto Wazuri" (Advice for a good life)<br>3. Counselling and guidance<br>Individual/Group	Wellbeing<br>Empowerment<br>Support	Refugee Camp<br>Interventions conducted by community members	20 UAMs<br>Burundi, Rwanda, Somalia, DRC<br>Focus group 1:<br>n=10 (6F), 12-14 years old<br>Focus group 2:<br>n=7 (4F), 15-18 years old<br>8 key informants<br>5 families (32-55 years old)	Focus group interviews<br>In-depth key interviews	Fewer developmental or behavioural problems, placement stability, safety, permanent contact with relatives, sharing of experiences, in most cases	Small sample size<br>Only participants from African countries<br>Not randomly recruited<br>Limited generalizability<br>No control group<br>No comparison with placebo or another intervention<br>No standardised measures of mental health and progress<br>No manual<br>No follow-up
Rogers et al. (2018), UK	Thematic analysis	Community based participatory research-Photovoice<br>Group<br>6 activity sessions<br>1 focus group with foster carers	Improvement of caregivers' skills	Collaboration between the university and a fostering service<br>Sessions conducted by social scientists	7 UAMs (M)<br>16-17 years old<br>Afghanistan, Albania<br>6 carers	Photographs<br>Focus group	Themes of UAM's images:<br>*Ordinary experiences in extraordinary circumstances*<br>*Building relationships*<br>Themes of Foster carers focus group:<br>*Concerns about meeting children's cultural, religious and linguistic needs*<br>*Concerns about radicalisation*<br>*Concerns about ages*<br>At the end of the photo discussions, the foster carers were open to attending further training	Small sample size<br>Only male participants from two countries<br>Not randomly recruited<br>Limited generalizability<br>No control group<br>No comparison with placebo or another intervention<br>Effects not demonstrated by different investigators<br>No standardised measures of mental health and progress<br>No manual<br>No follow-up
Case studies/series								
Magnano & Andrea (2019), Italy	Case study	Career Guidance<br>Individual/Group<br>7 meetings (6 in group setting, 1 in individual setting)	Integration<br>Wellbeing<br>Empowerment<br>Support	Host Community for UAMs<br>Sessions conducted by a career counsellor, with the support of a cultural mediator and/or educator	14 UAMs (M)<br>14-18 years old<br>Ivory Coast, Gambia, Senegal, Ghana, Guinea-Conakry, Republic of	Semi-structured interviews<br>3IP	Improvement of knowledge and interests regarding work, labour market, professions, change in definitions of work and decent work	Small sample size<br>Only male participants from African countries<br>Not randomly recruited<br>Limited generalizability<br>No control group<br>No comparison with placebo or

					Guinea		during intervention<br>At follow-up, positive influence of counselling only for the younger participants	another intervention<br>Effects not demonstrated by different investigators<br>No standardised measures of mental health and progress<br>No manual<br>Follow-up only with some participants<br>Possible effects of external factors at follow-up
Nelson et al. (2016), Australia	Case study	A model of social work with UAMs (critical social analysis, counselling, advocacy, social activism)<br>Individual	Wellbeing<br>Empowerment<br>Support	Community Detention house<br>Sessions conducted by a social worker	1 UAM (M)<br>15 years old<br>Afghanistan<br>Poor sleep, nightmares, anger, withdrawal, irregular school attendance, physical evidence of torture		Possible promotion of UAMs' wellbeing and reconnection of social workers with values of social justice and human rights<br>Potential for international transferability of the model	Results not generalizable because of single case study<br>Not randomly recruited<br>No research design<br>No control group<br>No comparison with placebo or another intervention<br>Effects not demonstrated by different investigators<br>No standardised measures of mental health and progress<br>No manual<br>No follow-up<br>No indication of specific results and effects of the intervention
Rania et al. (2018), Italy	Case study	Three new second-level models of intervention for the care of unaccompanied migrant minors:<br>1. "Safeguard Elfo"<br>2. "Apartments for Autonomy"<br>3. "Diffused Network"<br>Individual/Group	Improvement of caregivers' skills<br>Wellbeing<br>Empowerment<br>Support	<u>Involved agencies</u>: Regions, municipalities, courts, Defence for Children Italy, Third sector organizations, volunteer guardians and foster families, community centres, social services, foster families.	1. "Safeguard Elfo": 114 volunteer guardians, 7 UAMs<br>2. "Apartments for Autonomy": 366 UAMs and young adults<br>3. "Diffused Networks": UAMs and young adults, 9 male migrants and 3 mother-child dyads	Secondary data that the representatives of the intervention projects shared with the researchers<br>Semi-structured interviews with key informants (projects representatives, social workers, educators, volunteer guardians, and foster mothers)	Bringing the host population closer to minors<br>Developing individual autonomy	Undefined demographics and/or number of participants<br>Sample not randomly recruited<br>Limited generalizability<br>No research design<br>No control group<br>No comparison with placebo or another intervention<br>No standardised measures of mental health and progress<br>No manual<br>No follow-up
Tyminski (2018), Germany	Case study	An organizational consultation for an NGO that handles placements for UAMs<br>Group<br>1 day	Improvement of carergivers' skills	NGO that handles placements for UAMs<br>Consultation conducted by a mental health professional	Staff members of the NGO	Feedback from the staff	Identification of helpful mentoring strategies	Results not generalizable because of single case study<br>Not randomly recruited<br>No research design<br>No control group<br>No comparison with placebo or another intervention<br>Effects not demonstrated by different investigators<br>No standardised measures of mental health and progress<br>No manual<br>No follow-up

Practice papers								
Alem et al. (2021), Ethiopia		A multipronged approach for prevention, intervention, and postvention of suicidal incidents<br>Individual/Group<br>Psychoeducation: 1-3 sessions<br>Group counselling: 10 sessions<br>Individual counselling	Wellbeing<br>Empowerment<br>Support	Refugee Camp<br>Intervention conducted by selected counsellors with prior experience in working with children and trained staff members of an NGO	1174 minors<br>(398 F)<br>78% completed<br>≥7 group sessions<br>Eritrea	Feedback from the field	Reduction in psychological, somatic and/or behavioural symptoms of distress in 92.5% of those who completed the 6-month assessment	Participants only from one country<br>No age indicated<br>Not randomly recruited<br>Limited generalizability<br>No research design<br>No control group<br>No comparison with placebo or another intervention<br>No standardised measures of mental health and progress
Burck & Hughes (2018), UK		Psychosocial and therapeutic support (systemic–narrative practice and community/liberation psychology)<br>Individual/Group	Wellbeing<br>Empowerment<br>Support	Refugee Camp<br>Intervention conducted by systemic family therapists and clinical psychologists	UAMs, women, children and their families and many volunteers at the Calais camp	Feedback from the field	Witnessing, naming and acknowledging injustices<br>Promotion of solidarity	Undefined number and demographics of participants<br>Not randomly recruited<br>Limited generalizability<br>No research design<br>No control group<br>No comparison with placebo or another intervention<br>No standardised measures of mental health and progress<br>No manual<br>No indication of intervention duration<br>No follow-up
Clacherty (2015), South Africa		"The suitcase project"<br>An art-based project for war-affected children and young people who cross borders<br>Group	Wellbeing<br>Empowerment<br>Support	Initially an informal group run voluntarily by a child research practitioner and an artist	UAMs<br>9-17 years old<br>Burundi, Rwanda, DRC, Angola, Eritrea	Transcripts of the group discussions and artwork	Meaning making<br>Self-expression through art	Undefined number of participants<br>Participants only from African countries<br>Not randomly recruited<br>Limited generalizability<br>No research design<br>No control group<br>No comparison with placebo or another intervention<br>No standardised measures of mental health and progress<br>No indication of intervention duration<br>No follow-up
Estoura & Roberto (2019), Portugal	Practice proposal	The RAISE Model<br>Residential intervention for UAMs, with 5 pillars: Protection and Security; Attachment; Identity (ies); Participation; Citizenship<br>Individual/Group<br>5 months	Wellbeing<br>Empowerment<br>Support	Residential Facility<br>Intervention implemented by residential personnel (including mental health professionals) and external partners	UAMs	Unstructured and in-depth interviews<br>Observation by staff members		No indication of number, demographics and recruitment method of possible participants<br>No research design<br>No control group<br>No comparison with placebo or another intervention<br>No standardised measures of mental health and progress<br>No follow-up

Note: **3IP** = Iconographic Professional Interests Inventory, **CHS** = Children's Hope Scale, **CYRM-12** = Children and youth resilience measure, **DRC** = Democratic Republic of Congo, **F** = Female, **K10** = Kessler Psychological Distress Scale, **M** = Male, **MHCI** = Mental Health Capacity Instrument, **RSE** = Rosenberg Scale for Self-Esteem, **TABS** = Trauma Attachment Scale, **UAM** = Unaccompanied Minor, **YCWs** = Youth Care Workers

* $p < 0.05$, ** $p < 0.01$, *** $p < 0.001$
